# Sensitivity enhancement of nonlinear micromechanical sensors using parametric symmetry breaking

**DOI:** 10.1038/s41378-024-00784-4

**Published:** 2024-10-29

**Authors:** Yutao Xu, Qiqi Yang, Jiahao Song, Xueyong Wei

**Affiliations:** State Key Laboratory for Manufacturing Systems Engineering, Xi’an Jiaotong University, Xi’an, 710049 China

**Keywords:** Electrical and electronic engineering, Physics

## Abstract

The working mechanism of resonant sensors is based on tracking the frequency shift in the linear vibration range. Contrary to the conventional paradigm, in this paper, we show that by tracking the dramatic frequency shift of the saddle-node bifurcation on the nonlinear parametric isolated branches in response to external forces, we can dramatically boost the sensitivity of MEMS force sensors. Specifically, we first theoretically and experimentally investigate the double hysteresis phenomena of a parametrically driven micromechanical resonator under the interaction of intrinsic nonlinearities and direct external drive. We demonstrate that the double hysteresis is caused by symmetry breaking in the phase states. The frequency response undergoes an additional amplitude jump from the symmetry-breaking-induced parametric isolated branch to the main branch, resulting in double hysteresis in the frequency domain. We further demonstrate that significant force sensitivity enhancement can be achieved by monitoring the dramatic frequency shift of the saddle-node bifurcations on the parametric isolated branches before the bifurcations annihilate. Based on the sensitivity enhancement effect, we propose a new sensing scheme which employs the frequency of the top saddle-node bifurcation in the parametric isolated branches as an output metric to quantify external forces. The concept is verified on a resonant MEMS charge sensor. A sensitivity of up to 39.5 ppm/*f*C is achieved, significantly surpassing the state-of-the-art resonant charge sensors. This work provides a new mechanism for developing force sensors of high sensitivity.

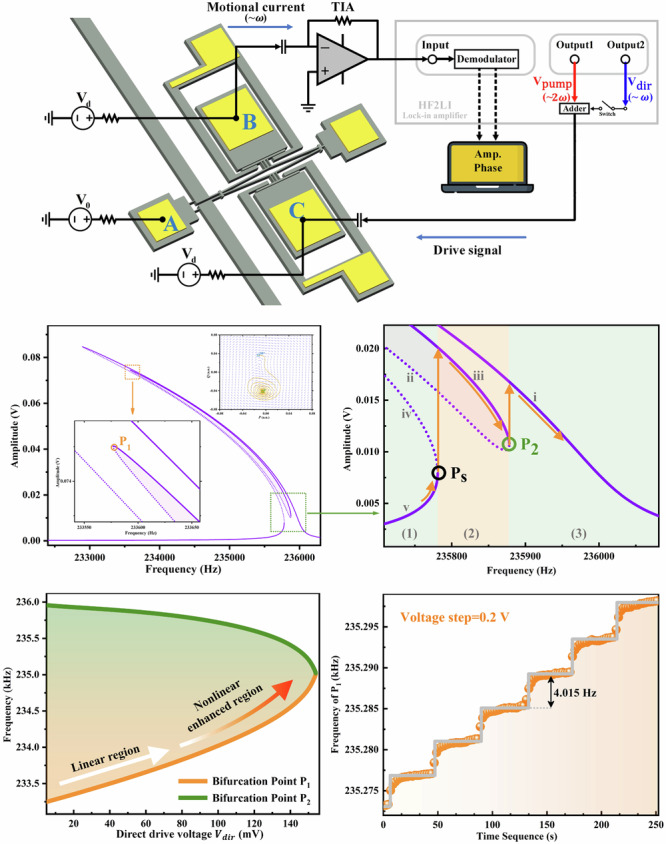

## Introduction

Due to their low cost, high sensitivity, and wide dynamic range, MEMS resonant sensors have been utilized in many fields, e.g., inertial navigation, seismic detection, and consumer electronics^1,2^. Conventionally, the working mechanism of resonant sensors is based on tracking the frequency shift in the linear vibration range. However, there are many working conditions, such as environmental thermal noise^3,4^, circuit component interference^5^, and electrical dissipation^6^, that severely influence the enhancement of signal-to-noise ratio, degrading the resolution of these sensors operating in the linear regime. Enhancing the signal-to-noise ratio by increasing driving intensity can improve the sensing resolution according to Robins’ formula^7,8^, provided that the sensor remains in the linear regime. However, with the dimensions of resonators scaled down, nonlinear effects become dominant at low amplitude, which causes severe amplitude to frequency conversion and leads to degradation in frequency stability. Therefore, optimization of sensing performance is typically achieved by increasing the quality factor without considering the strategies for signal-to-noise ratio enhancement^9–12^.

In recent years, nonlinear effects such as parametric coupling^13,14^, internal resonance^15–18^, synchronization^19–22^, and nondegenerate parametric resonance^23–26^ have been found to significantly improve the performance of oscillators and resonant sensors. Achieving better performance in the presence of nonlinearity has attracted wide interest. Antonio et al. achieved significant frequency stabilization of the fundamental mode by coupling it to a higher-order mode through 1:3 internal resonance^15^. Intense energy exchange suppresses the amplitude noise as well as the frequency fluctuation of the fundamental mode. Agrawal et al. reported on observations of internal synchronization in coupled micromechanical oscillators and demonstrated a significant improvement in the frequency stability of synchronized micromechanical oscillators^20^. Wang et al. demonstrated that the internal resonance between two electrostatically coupled micromechanical resonators with a frequency ratio of 1:3 can improve the frequency stability by seven-fold, resulting in a detection resolution of a single electron^17,27^. Although several proposals have been implemented to improve frequency stability and sensing resolution with nonlinear coupling, they require specific operating conditions such as frequency commensuration, thus taking on new challenges to the design of micro-devices and self-oscillation sustaining circuits^28^.

Degenerate nonlinear parametric resonance of resonators, due to its features of signal amplification^29^, noise squeezing^30^, and frequency stabilization^31^, plays a vital role in many areas of science and technology. Sensing applications of parametric resonance, including parametric mass sensors^32–34^, parametric gravitational waves detection^35^, parametric gyroscopes^36–38^, and parametric symmetry breaking transducers^39–42^, show unique advantages over conventional sensing paradigms. Among the sensing mechanisms based on parametric resonance, parametric symmetry breaking has attracted substantial interest. Symmetry breaking occurs when an external force is injected into the parametrically driven resonator. The external force manipulates the double well potential and breaks the symmetry between two parametric phase states, which can potentially be utilized to extend the detection limit of microelectromechanical resonant sensors. For example, Papariello et al. experimentally observed the double hysteresis caused by symmetry breaking of parametrically driven resonators for the first time^40^. A hysteretic force sensing scheme based on the linear frequency shift in the double hysteresis region is proposed and validated through numerical simulation^41^, which can potentially realize force detection in the attonewton (aN) range. However, the existing works on symmetry breaking only use the linear frequency shift of the second jump in the double hysteresis response, and the double hysteresis no longer exists under a small external perturbation which will limit the dynamic range of sensors. In addition, the sensing scheme based on symmetry breaking has not been experimentally demonstrated, and only the theoretical predictions exist in the literature. Therefore, it is worthy of exploring the symmetry breaking phenomena in closed-loop experiments and verifying its potential in sensing applications.

Towards these objectives, in this work, we introduce a new sensing scheme with enhanced sensitivity that relies on the nonlinear shrinking of the parametric isolated branch under external perturbation. The same number of external stimuli induces a more remarkable frequency shift than that in the sensing paradigm proposed by Papariello et al^41^. Specifically, we first theoretically and experimentally explore the double hysteresis behaviors of the parametrically driven micromechanical resonator under the combination of the parametric excitation and direct external drive. We theoretically map the phase portraits to reveal the cause of double hysteresis and determine the stabilities of the theoretical solutions. In addition, we theoretically demonstrate that significantly enhanced force sensitivity can be achieved by operating the nonlinear parametric sensor in the top bifurcation of the parametric isolated branch and monitoring the dramatic frequency shift of the bifurcation under external force. The proposed sensitivity enhancement scheme is validated by implementing a closed-loop experiment of charge detection.

This paper is organized as follows. The Introduction section gives a brief introduction to MEMS resonant sensors and their shortcomings. The Results section is divided into three subsections. Firstly, the *device and characterization* subsection shows an overview of the device under test and the basic dynamic features of the micromechanical resonator. Secondly, in the *double hysteresis phenomena and parametric study* subsection, the double hysteresis of the parametrically driven system under perturbation is experimentally demonstrated, and the symmetry breaking of the phase states under direct external excitation is theoretically verified to be the cause of double hysteresis. Moreover, we implement a parametric study on the double hysteresis both experimentally and theoretically. Thirdly, in the *sensitivity enhancement scheme and its experimental demonstrations* subsection, a new sensitivity enhancement scheme is proposed based on the symmetry breaking phenomena, and the scheme is verified by conducting real-time charge detection. The Discussion section summarizes the limitations of the proposed sensing scheme. The Conclusion section summarizes the contributions of this paper and suggests avenues for future works.

## Results

### Device and characterization

A diamond-shaped MEMS resonator consisting of two identical arch beams is considered in this work. We specifically designed our device to facilitate the observation of dynamic characteristics in nonlinear regimes, which is achieved by the diamond-shaped structure and large capacitance area. The diamond-shaped structure allows the resonator to exhibit a negative Duffing-like response; see Supplementary Section 3 for detailed structural parameters.

**Fig. 1:**
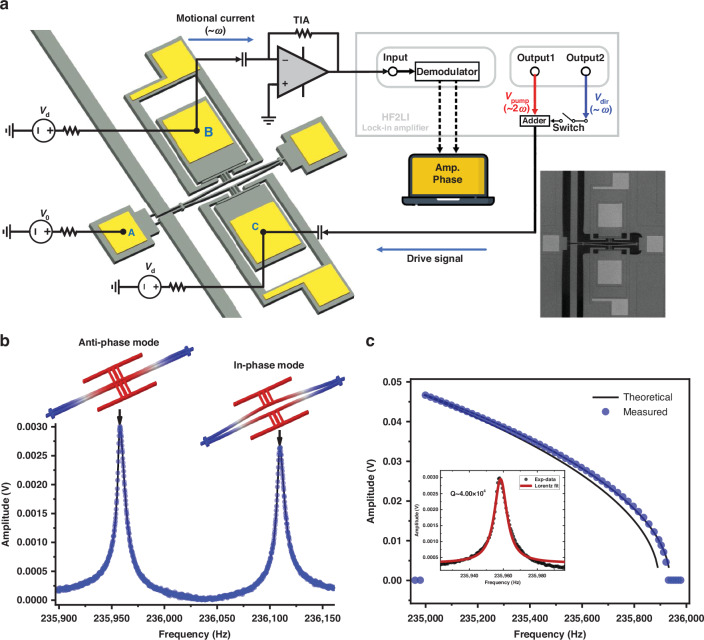
Characterization of the device under test. **a** The diamond-shaped MEMS resonator was pumped, driven, and sensed by capacitive electrodes labelled from A to C. The DC bias ${V}_{0}=0$ was applied on electrode A to ground the resonator body. **b** The linear amplitude-frequency response and the corresponding mode shapes of the first-order anti-phase and in-phase flexural modes. The resonance curve was obtained under only the direct external drive, with ${V}_{{dir}}$ being set to 5 mV and ${V}_{d}$ being set to 30 V. The anti-phase mode with lower resonant frequency was involved in this study. **c** Experimental and theoretical nonlinear parametric resonance curves of the device with a parametric pump voltage of ${V}_{{pump}}=1\text{V}$ and a DC driving voltage of ${V}_{d}=30\text{V}$. The red solid line in the inset is the Lorentz fit of the measured linear amplitude response. The quality factor of the resonator was measured as 4.00$\times {10}^{4}$ utilizing the half-power bandwidth method

A schematic of the experimental setup used for testing is shown in Fig. 1a. All measurements were conducted in a vacuum chamber with a pressure level under ${10}^{-1}$ Pa. A low noise power supply was used to apply the desired DC driving voltage signal ${V}_{d}$ and DC bias ${V}_{0}$. The DC bias ${V}_{0}=0$ was applied to electrode A, thereby grounding the resonator body. The parametric pump voltage ${V}_{{pump}}$ with a frequency near twice the resonant frequency (denoted by 2$\omega$) and the direct external drive voltage ${V}_{{dir}}$ with a frequency near the resonant frequency (denoted by $\omega$) were generated through the Output1 port and Output2 port of the HF2LI lock-in amplifier, respectively. When the switch is closed, the direct external drive and parametric pump can be injected into the resonator body simultaneously or separately through electrode C utilizing the built-in adder of the lock-in amplifier. The phase difference between the two signals can be controlled by the host computer. The capacitive actuation method was utilized across the 3.0 μm gap to characterize the dynamic features of the first-order anti-phase flexural mode in the frequency domain. The resonator was electrostatically excited by applying the combined voltage of $V(t)={V}_{{ac}}+{V}_{d}$ to electrode C, where ${V}_{{ac}}$ is equal to ${V}_{{pump}}+{V}_{{dir}}$ and ${V}_{d}$ is the DC driving voltage. The motion of the resonator was detected via the capacitive sensing method. The DC voltage ${V}_{d}$ applied to the electrode B was used to facilitate the capacitive sensing method (see Supplementary Section 3 for details). The motional current representing the magnitude of the mechanical motion was amplified by the trans-impedance amplifier (TIA) and converted into a voltage signal. The amplified signal was then demodulated by the built-in demodulator in the lock-in amplifier to extract the amplitude and phase. See Supplementary Section 3 for the detailed experimental setup.

As shown in the dot-line of Fig. 1b, we measured the linear response of the doubly clamped diamond-shaped MEMS resonator under only the direct external drive. The resonator exhibits an anti-phase flexural mode with a resonant frequency ${\omega }_{0}$ of 2${\pi}\times$235.950 kHz and an in-phase flexural mode with a resonant frequency ${\omega }_{1}$ of 2${\pi}\times$236.109 kHz. In addition, we measured the nonlinear parametric response of the anti-phase flexural mode under study. Parametric excitation was realized by periodic modulation of the linear stiffness near twice the resonant frequency through the electrostatic softening effect. Unlike the Duffing oscillator, the frequency of the diamond-shaped resonator decreases with the increase of the vibration amplitude, showing a significant stiffness softening effect (Fig. 1c). The black line is the theoretical resonance curve implemented in Supplementary Section 1 using the fitting parameters of the resonator. The quality factor of the resonator was measured as 4.00$\times {10}^{4}$ from its linear frequency response under a small direct external drive utilizing the half-power bandwidth method, as shown in the inset of Fig. 1c. The black dot line is the experimental data and the red solid line is the Lorentz fit.

### Double hysteresis phenomena and parametric study

**Fig. 2:**
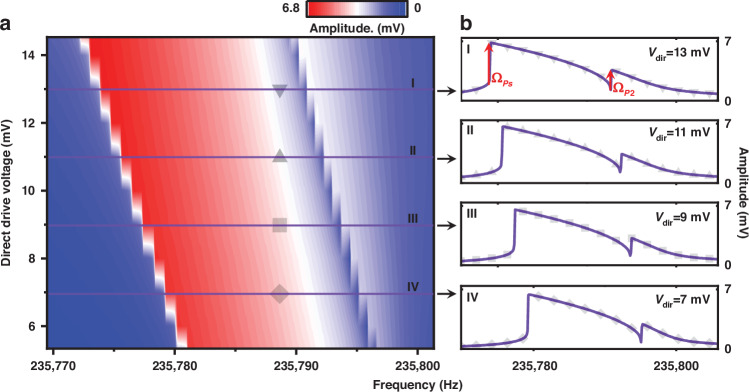
Double hysteresis phenomena. **a** Double hysteresis response under the combined excitation of parametric pump voltage (2.0 V), direct external drive voltage (range from 5 to 15 mV), and DC bias (40 V). The phase difference between the parametric pump and direct external drive was set to −15 degrees during the experiment. **b** Some slices of amplitude-frequency response in (**a**), which are donated by number I-IV. The red arrows indicate the direction of the amplitude jumps

Next, we investigated the double hysteresis phenomena under the combined excitation of the parametric pump and direct external drive. The MEMS resonator in Fig.1a was parametrically driven by a parametric pump ( ~ $2{\omega }_{0}$), while a direct external drive ( ~ ${\omega }_{0}$) was simultaneously applied on the resonator body with the signal adder. We monitored the frequency response at frequency ~${\omega }_{0}$, and the results with different direct external drive voltages ${V}_{{dir}}$ are shown in Fig. 2a. The parametric pump voltage ${V}_{{pump}}$ and DC bias voltage were maintained at 2 V and 40 V, respectively, and the direct external drive voltage was increased from 5 to 15 mV in steps of 1 mV. The double hysteresis occurs in the upward frequency sweeping, which is similar to a nonlinear Duffing response accompanied by an amplitude pit. Some slices of the frequency response curves are plotted in Fig. 2b to better illustrate the evolution of double hysteresis with the direct external drive. The red arrows in Fig. 2b represent the direction of the two consecutive amplitude jumps during the upward frequency sweep. The first amplitude jump at frequency ${\varOmega }_{{Ps}}$ describes the typical hysteresis for a parametrically driven resonator with negative Duffing-like nonlinear stiffness, while the second amplitude jump at frequency ${\varOmega }_{P2}$ is a new feature that occurs under the interaction between the parametric pump and direct external drive. The frequency of the amplitude pit, i.e., the frequency of the second jump point (${\varOmega }_{P2}$), decreases linearly with the increase of the direct external drive.

To understand the unique behavior of the double hysteresis in our nonlinear parametric system, we formulate the mathematical model for the parametrically driven resonator utilizing the Euler-Bernoulli beam theory and Kevin-Voigt theory while accounting for the geometric, electrostatic, and damping nonlinearities:

1 $$\begin{array}{l}\left(\rho A+{m}_{c}\delta \left(\widetilde{x}-\frac{\widetilde{l}}{2}\right)\right)\frac{{\partial }^{2}{\tilde{\mathcal{w}}}}{\partial {\widetilde{t}}^{2}}+{EI}\frac{{\partial }^{4}{\tilde{\mathcal{w}}}}{\partial {\widetilde{x}}^{4}}+\widetilde{C}\frac{\partial {\tilde{\mathcal{w}}}}{\partial \widetilde{t}}+\xi I\frac{{\partial }^{5}{\tilde{\mathcal{w}}}}{\partial \widetilde{t}\partial {\widetilde{x}}^{4}}\\-\frac{{EA}}{2\widetilde{l}}\left[\frac{{\partial }^{2}{\tilde{\mathcal{w}}}}{\partial {\widetilde{x}}^{2}}+\frac{{\partial }^{2}{{\tilde{\mathcal{w}}}}_{0}}{\partial {\widetilde{x}}^{2}}\right]{\int_{0}^{\widetilde{l}}}\left[{\left(\frac{\partial {\tilde{\mathcal{w}}}}{\partial \widetilde{x}}\right)}^{2}+2\left(\frac{\partial {{\tilde{\mathcal{w}}}}_{0}}{\partial \widetilde{x}}\frac{\partial {\tilde{\mathcal{w}}}}{\partial \widetilde{x}}\right)\right]d\widetilde{x}\\-\frac{\xi A}{2\widetilde{l}}\left[\frac{{\partial }^{2}{\tilde{\mathcal{w}}}}{\partial {\widetilde{x}}^{2}}+\frac{{\partial }^{2}{{\tilde{\mathcal{w}}}}_{0}}{\partial {\widetilde{x}}^{2}}\right]{\int_{0}^{\widetilde{l}}}\left[2\left(\frac{{\partial }^{2}{\tilde{\mathcal{w}}}}{\partial \widetilde{t}\partial \widetilde{x}}\frac{\partial {\tilde{\mathcal{w}}}}{\partial \widetilde{x}}\right)+2\left(\frac{\partial {{\tilde{\mathcal{w}}}}_{0}}{\partial \widetilde{x}}\frac{{\partial }^{2}{\tilde{\mathcal{w}}}}{\partial \widetilde{t}\partial \widetilde{x}}\right)\right]d\widetilde{x}\\=\widetilde{\varLambda }\left({\tilde{\mathcal{w}}},\widetilde{\varOmega }\widetilde{t}\right)\delta \left(\widetilde{x}-\frac{\widetilde{l}}{2}\right)+\widetilde{{\rm{H}}}\left(\widetilde{\varOmega }\widetilde{t}\right)\delta \left(\widetilde{x}-\frac{\widetilde{l}}{2}\right)\end{array}$$

where ${\tilde{\mathcal{w}}}$ represents the transverse deflection of the resonator, ${{\tilde{\mathcal{w}}}}_{0}$ represents the microbeam profile, $\rho$ is the density, $A$ is the area of the cross-section of the microbeam, ${m}_{c}$ is the concentrated mass in the middle, $\widetilde{x}$ is the position along the microbeam length, $\widetilde{l}$ is the length of the microbeam, $E$ is the Young’s modulus, $\widetilde{C}$ is the linear damping per unit length, $\xi$ is the viscous damping coefficient, $I$ is the moment of inertia of the cross section, parametric excitation $\widetilde{\varLambda }\left({\tilde{\mathcal{w}}},\widetilde{\varOmega }\widetilde{t}\right)$ is a function of ${\tilde{\mathcal{w}}}$ and $\widetilde{\varOmega }\widetilde{t}$, direct external drive $\widetilde{{\rm H}}\left(\widetilde{\varOmega }\widetilde{t}\right)$ is a function of $\widetilde{\varOmega }\widetilde{t}$, $\widetilde{t}$ is the time, $\widetilde{\varOmega }$ is the driving frequency, and $\delta$ is the Dirac function. Utilizing Galerkin projection, the dimensionless reduced-order governing equation is obtained as

2 $$\ddot{u}+\frac{1}{Q}\dot{u}+\left(1-\mathrm{\lambda \; cos}\left(2\omega \tau \right)\right)u+{\alpha u}^{2}+{\beta u}^{3}+{\eta u}^{2}\dot{u}={hcos}(\omega \tau +{\varphi }_{0})$$

where the dot overhead symbol denotes differentiation with respect to the dimensionless time $\tau$, $u$ is the dimensionless amplitude, $Q$ is the quality factor, $\lambda$ is the dimensionless parametric pump produced by ${V}_{{pump}}$, $\omega$ is the dimensionless driving frequency, $\alpha$ is the dimensionless quadratic nonlinear coefficient caused by the arch structure, $\beta$ is the dimensionless Duffing coefficient, $\eta$ is the dimensionless nonlinear damping coefficient, $h$ is the dimensionless direct external force produced by ${V}_{{dir}}$, and ${\varphi }_{0}$ is the phase difference between the parametric pump and direct external drive. More detailed analysis for mathematical modelling and the perturbation analyses are presented in Supplementary Section 1.

**Fig. 3:**
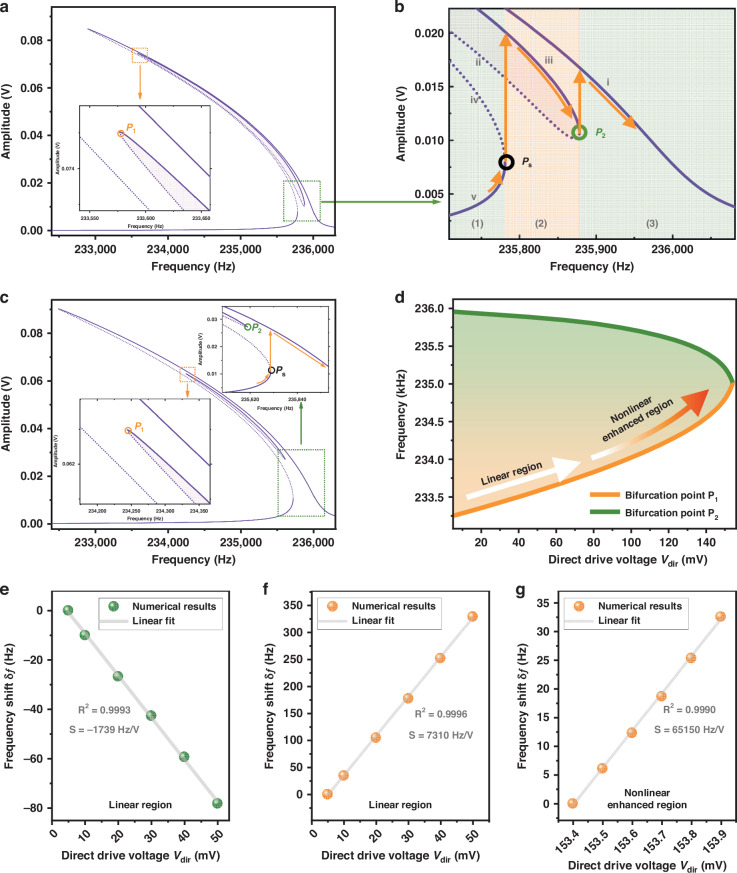
Numerical simulations and parametric study. **a** Shows the numerical amplitude-frequency curve under the combined excitation of the parametric pump voltage (2 V) and direct external drive (0.05 V). The DC bias and the phase difference are set to 40 V and 45 degrees, respectively. **b** Zoom-in of (**a**). **c** shows the numerical amplitude-frequency curve of the system when the direct external drive is increased to 0.12 V. All other parameters are constant with those in (**a**). **d** The relationship between the bifurcation frequencies and the intensity of the direct external drive. **e** The linear dependence of ${\varOmega }_{P2}$ on the direct external drive. **f**, **g** Show the enhanced sensitivity of the top bifurcation frequency ${\varOmega }_{P1}$ on the direct external drive when the resonator is navigated to the nonlinear enhanced region

Figures 3a–c show the theoretical frequency responses of the parametrically driven resonator which show good agreement with the experimental data in Fig. 2a by reproducing all the features. The calculated dimensionless responses are rescaled according to the transformation coefficients (see Supplementary Section 1 for details). The stabilities of the theoretical solutions are determined through the basins of attraction (see the *stability analysis* subsection in the section of “Materials and methods”). The solid line and dotted line represent the stable and unstable solutions, respectively. Specifically, as shown in Fig. 3a, b, for the small external drive (${V}_{{dir}}$ = 0.05 V), owing to the interaction of the parametric pump and direct external excitation, a set of separate branches ii and iii emerge in addition to the main negative Duffing-like response branches i and iv. These separate branches, which we term “parametric isolated branches” for the rest of this paper, are defined in Fig. 3b. The parametric isolated branches are surrounded by the main branches, exhibiting two extreme bifurcation frequencies: ${\varOmega }_{P1}$ at the top saddle-node bifurcation ${P}_{1}$ and ${\varOmega }_{P2}$ at the bottom saddle-node bifurcation ${P}_{2}$. Since the bottom bifurcation ${P}_{2}$ lies to the right of bifurcation ${P}_{s}$, the response obtained by upward frequency sweep exhibits two consecutive jumps at the boundaries between domain (1) and domain (2) and between domain (2) and (3), respectively. The amplitude response jumps from the stable isolated branch iii to the main branch i, and the vibrational phase state will undergo a reversal at the bottom saddle-node bifurcation ${P}_{2}$, resulting in a double hysteresis in the amplitude response.

As the direct external drive increases to 0.12 V, the isolated branches further shrink, as shown in Fig. 3c, causing the bifurcation frequencies ${\varOmega }_{P1}$ and ${\varOmega }_{P2}$ to move closer. Since the bottom bifurcation ${P}_{2}$ lies to the left of bifurcation ${P}_{s}$, only the first jump at bifurcation ${P}_{s}$ occurs during upward frequency sweep (indicated by the orange arrows), in which case the frequency response only exhibits typical negative Duffing characteristics without double hysteresis. We extract the frequencies of bifurcations ${P}_{1}$ and ${P}_{2}$ under different direct external drives, as shown in Fig. 3d. The two bifurcations gradually approach and eventually annihilate. Figure 3e shows the linear dependence of the second jump frequency ${\varOmega }_{P2}$ on ${V}_{{dir}}$ with a sensitivity of 1739 Hz/V, which is qualitatively consistent with the experimental results in Fig. 2a, b. As shown in Fig. 3f, g, the sensitivity of the top bifurcation frequency ${\varOmega }_{P1}$ increases from 7310 Hz/V (in the linear region) to 65,150 Hz/V when the device is operated in the nonlinear enhancement region, which offers valuable implications in designing high-performance force-based sensors.

**Fig. 4:**
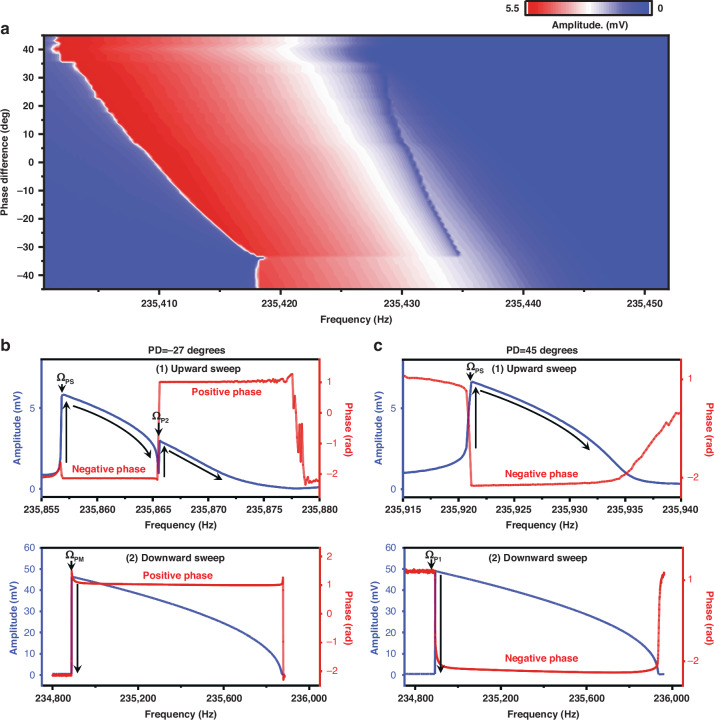
Dependence of double hysteresis on phase difference. **a** Dependence of double hysteresis on phase difference. The responses were measured under the combined excitation of parametric pump voltage (2.0 V), direct external drive voltage (5 mV), and DC bias (40 V). **b** and **c** plot the amplitude-frequency responses and phase-frequency responses obtained by upward and downward frequency sweeps under two representative phase differences

In addition, we further analyze the dependence of double hysteresis on the phase difference (PD) ${\varphi }_{0}$ between the parametric pump and direct external drive. PD leads to two qualitatively different behaviors, as will be shown below. Figure 4a shows the measured responses under different PDs obtained by upward frequency sweep. It can be observed that the double hysteresis phenomena exist only in the PD range of -33 to 32 degrees. The frequency of the second jump ${\varOmega }_{P2}$ depicts a nonlinear dependence on the PDs. Figures 4b and c show the response curves under two representative PDs. The black arrows represent the direction of the amplitude jumps and frequency sweep. At the PD of −27 degrees, the double hysteresis occurred during the upward frequency sweep (Fig. 4b top). The response phases corresponding to the parametric isolated branch and the main branch are negative and positive, respectively^40^. At the bottom saddle-node bifurcation ${P}_{2}$, the response switches from the isolated branch to the main branch, and the phase state reverses, which results in double hysteresis in the response curve. However, during the downward frequency sweep at PD of −27 degrees, the measured displacement amplitude traverses the main branch without passing through the isolated branch, as shown in the bottom half of Fig. 4b. When the PD was increased to a specific value beyond the upper limit of the PD range in which double hysteresis occurs (e.g., 45 degrees in this experimental setup), the measured amplitude-frequency response curves pass through the parametric isolated branch in both upward and downward frequency sweeps (inferred from the negative response phase as shown in Fig. 4c). The response amplitude of the nonlinear parametric resonance under the downward frequency sweep increases with decreasing frequency until it hits the top saddle-node bifurcation ${P}_{1}$ and falls.

### Sensitivity enhancement scheme and its experimental demonstrations

As evident in the previously discussed results, a sustained increase in direct external drive leads to an intensification of symmetry breaking, which can effectively improve the frequency sensitivity of the bifurcations ${P}_{1}$ and ${P}_{2}$ to the external perturbation. As shown in Fig. 3d, bifurcation frequencies ${\varOmega }_{P1}$ and ${\varOmega }_{P2}$ first vary linearly with ${V}_{{dir}}$ in the linear region, which is in line with that observed by Papariello et al^41^.; however, further increasing ${V}_{{dir}}$ will induce a nonlinear frequency shift of the saddle-node bifurcations in the nonlinear enhanced region. Based on the significant shrink of the parametric isolated branches under external perturbation in the nonlinear enhanced region, we propose a new sensing scheme that exploits the enhanced frequency shift of the top bifurcation frequency ${\varOmega }_{P1}$ to external perturbation. The sensing scheme uses the top bifurcation frequency ${\varOmega }_{P1}$ as the output metric to quantify the external forces.

**Fig. 5:**
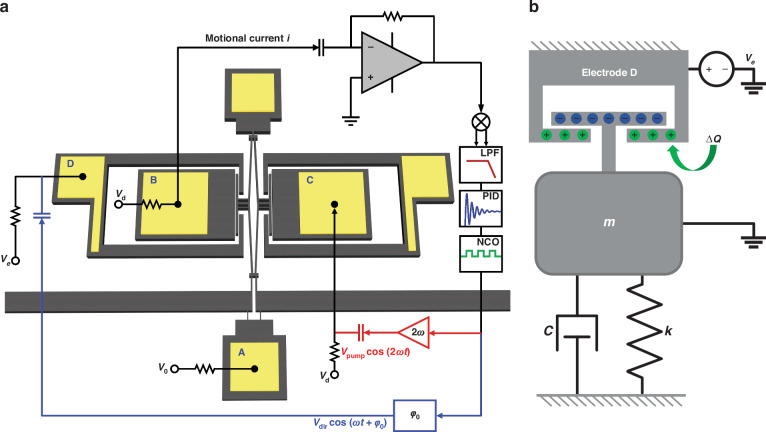
Schematic of the closed-loop measurement circuit. **a** The close-loop circuit for the real-time charge detection. The charge sensor generates stable self-sustained oscillation utilizing a built-in parametric phase-locked loop in Zurich instruments HF2LI lock-in amplifier; LPF: low-pass filter; PID: proportional integral derivative; NCO: numerically controlled oscillator. Phase control is realized by adjusting the phase setpoint in the PID module. **b** A spring-damper-mass model for describing the perturbation applied to the charge sensor produced by the charge input on the capacitor

To experimentally validate the sensitivity enhancement mechanism, we implemented an experimental demonstration of real-time charge detection utilizing the MEMS resonator shown in Fig. 1a. The closed-loop circuit of the MEMS charge sensor is shown in Fig. 5a. A parametric phase-locked loop was set up for holding the oscillations at the top bifurcation point ${P}_{1}$. In a parametric phase-locked loop, we first monitor the vibration of the resonator using the capacitance method. The amplified voltage signal representing the vibrational motion is then fed into the lock-in amplifier. The amplitude and the phase of the motion are demodulated in the built-in demodulator of the lock-in amplifier. The demodulated phase information is first low-pass filtered and then fed into the PID module of the parametric phase-locked loop, which is utilized to calculate the phase error with respect to the set point of the phase. The phase error is utilized to adjust the frequency of the NCO (numerically controlled oscillator) so as to minimize the phase error and maintain the stable oscillation of the charge sensor at the set point of phase. The frequency of the NCO is amplified and frequency doubled to provide the desired parametric pump voltage ${V}_{{pump}}$ at the frequency of 2$\omega$. For the direct external drive voltage ${V}_{{dir}}$, the frequency of the NCO is amplified to provide the desired ${V}_{{dir}}$ at the frequency of $\omega$. The phase difference ${\varphi }_{0}$ between ${V}_{{pump}}$ and ${V}_{{dir}}$ can be adjusted on the host computer. A DC voltage ${V}_{e}$ is applied on electrode D to generate and maintain known quantities of charge.

Before implementing charge sensing, in order to initialize the charge sensor, we first drive the resonator with a pure parametric pump (${V}_{{pump}}=800\text{mV}$, ${V}_{d}=40\text{V}$, ${V}_{{dir}}=20\text{mV}$, ${V}_{e}=0\text{V}$, and ${\varphi }_{0}=45^{\circ}$), in which case the perturbation caused by the direct external drive equals zero. The resonator is equally likely to initiate parametric vibrations at either of the two phase states separated by ${\pi}$ radians^43^. After the parametric resonator initiates parametric vibration at the response branch with a negative phase response, we use the parametric phase-locked loop to make the charge sensor generate stable oscillation. The phase setpoint of the parametric phase-locked loop is then manually adjusted so as to navigate the charge sensor to the peak amplitude. This response branch with negative phase response is the “parametric isolated branch” before shrinking so that the charge sensor generates stable oscillations at the top bifurcation ${P}_{1}$. After the charge sensor generates stable oscillations at bifurcation ${P}_{1}$, input charge $\Delta Q$ is injected into the sensor through the input electrode D.

The schematic of the charge sensor is shown in Fig. 5b. The input charge $\Delta Q$ collected at electrode D will cause a change in the voltage ${V}_{e}$ in the form of $\Delta {V}_{e}=\Delta Q/C$, where $C$ = 17.48549 *f*F is the capacitance value of the parallel capacitor plate consisting of the resonator and the input electrode D (calculated by finite element simulation). Since the strength of the direct external drive is proportional to ${V}_{e}{V}_{{dir}}$ in the closed-loop measurement circuit, this change in ${V}_{e}$ will break the symmetry between the two-phase states of the parametrically driven MEMS sensor in the form of ${V}_{e}{V}_{{dir}}\mathrm{cos}(\omega t)$, allowing for the shrink (positive charge) or expansion (negative charge) of the parametric isolated branches. A significant shift in the frequency of the top bifurcation ${P}_{1}$ will occur, which can be utilized to accurately detect the input charge by monitoring the oscillation frequency of the charge sensor.

**Fig. 6:**
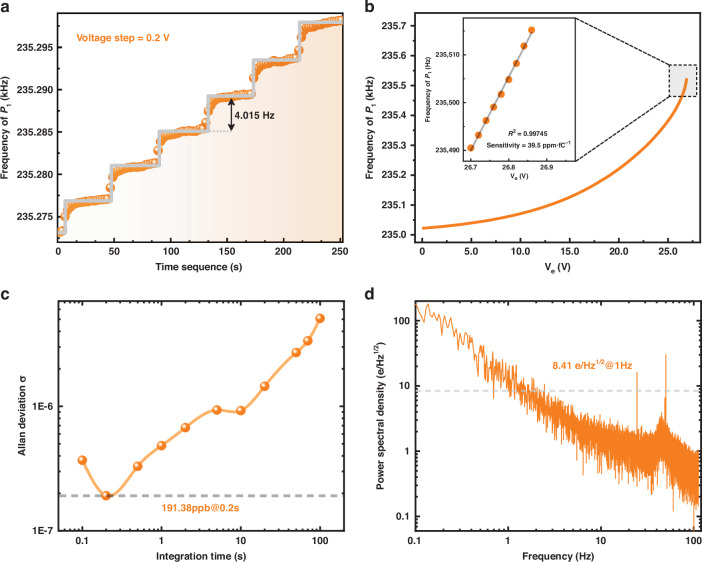
Charge detection in the closed-loop configuration. **a** The frequency of the top bifurcation point ${P}_{1}$ shifted stepwise with the increase of DC voltage ${V}_{e}$. **b** Measurement sensitivity calibration. With the accumulation of input charge, the measurement sensitivity enhancement was observe**d**. **c** and **d** show the Allan deviation and power spectral density of the nonlinear parametric sensors at the top bifurcation point ${P}_{1}$, respectively

For calibration of charge sensitivity, we applied known quantities of charge on electrode D utilizing a high-voltage power supply (DC voltage ${V}_{e}$ shown in Fig. 5a). The quantities of the input charge can be determined by the multiplication of voltage ${V}_{e}$ and the capacitance value *C* of the parallel capacitor plate. Figure 6a shows the real-time detection ladder diagram as the DC voltage ${V}_{e}$ varies in steps of 0.2 V. We observed the frequency shift of the MEMS sensor in real-time to the charge accumulation. The grey solid line corresponds to the average bifurcation frequency ${\varOmega }_{P1}$ at each detection ladder. The bifurcation frequency ${\Omega }_{P1}$ is shown in Fig. 6b as the voltage ${V}_{e}$ was varied in step from 0 V to 26.9 V. Significant enhancement in measurement sensitivity was clearly observed with the increase of ${V}_{e}$, a sensitivity of 6.33 ppb/e (39.5 ppm *f*C^-1^) was achieved when the charge sensor was operated in the nonlinear enhanced region (${V}_{e} \sim$26.7 V), which is six times better than that at the linear region (6.39 $\text{ppm}\cdot {f\text{C}}^{-1}$).

The detection limitation (resolution $R$) of the MEMS sensor depends on both measurement sensitivity $S$ and minimum detectable frequency shift ${\delta f}_{\min }$ with an expression of $R=C\cdot {\delta f}_{\min }/S$. In order to acquire ${\delta f}_{\min }$, oscillation frequency was collected for a duration of 300 s to calculate the Allan deviation ${\sigma }_{A}(\tau )$. The minimum Allan deviation reaches 0.19138 ppm at an integration time of 0.2 s, as shown by the orange dotted line in Fig. 6c. Therefore, the minimum detectable frequency shift ${\delta f}_{\min }$ can be estimated by the product of minimum Allan deviation and oscillation frequency (${\delta f}_{\min }={\sigma }_{A}\left(\tau \right)\cdot {f}_{{osc}}$), resulting in an electron resolution of 30.24 electrons. It is worth noting that the potential of our sensing paradigm is not fully revealed due to the limitation of the frequency tracking capability of our phase-locked loop. An electron resolution of 7.34 electrons can be obtained at the expense of frequency tracking capability (see Supplementary Section 4 for details). The noise power spectral density of the charge sensor is acquired by fast Fourier transformation analysis of the measured frequency. As shown in Fig. 6d, the noise floor of the charge sensor is 8.41 e/${\text{Hz}}^{1/2}$ at 1 Hz.

Table S3 in Supplementary Section 5 lists comparisons of the state-of-the-art charge sensors that use the micromechanical resonators as the core elements in terms of mechanism, footprint, charge resolution, and sensitivity. The sensor in this work has a charge sensitivity of 39.5 ppm/*f*C, exceeding the best reported result of 22.531 ppm/*f*C when compared to other counterparts of resonant charge sensors. In comparison, the charge sensor in this work achieves a moderate resolution. However, the potential of our sensing scheme is not fully realized due to the limitations of the phase-locked loop circuit, and the proposed sensing scheme in this work can be readily incorporated into a variety of other resonant sensors used for weak force sensing without the need for additional modes or auxiliary structural design.

## Discussion

In this paper, we designed a diamond-shaped micromechanical double-clamped beam and investigated the symmetry breaking of parametric phase states exhibited by the parametrically driven micromechanical resonator. We explored the use of parametric symmetry breaking to achieve high measurement sensitivity. The sensing scheme achieves the highest charge sensitivity compared with the resonant charge sensors using frequency as the output metric reported in recent years. Compared with the traditional sensing mechanism based on nonlinear effects, our scheme does not require an auxiliary resonator or mode, which provides a high-performance sensing paradigm with high robustness.

However, the potential of our sensing scheme is not fully realized. The charge sensor in this work achieves a moderate charge resolution, and the enhanced sensitivity is realized at the expense of dynamic range. This moderate charge resolution may be attributed to the large frequency drift caused by the high temperature coefficient of the single crystal silicon and the limitations on the frequency tracking ability of the commercial phase-locked loop. Firstly, our silicon-based resonant sensor severely suffers from the large frequency drift, which results in the deterioration of the eventual sensing resolution. As depicted in Fig. 6c, the significant temperature drift causes the Allan deviation to be proportional to ${\tau }^{1}$ when the integration time is over 0.2 s, which indicates a severe temperature-frequency conversion. Passive and active temperature compensation approaches such as composite structures, doping, geometry engineering, electronic compensation, and micro-oven control can be utilized to moderate the temperature drift and improve the measurement resolution in future work. Secondly, since the frequency tracking ability of the phase-locked loop depends on its bandwidth, the larger the bandwidth the stronger the frequency tracking ability. The significant frequency variation due to the large sensitivity of our charge sensor makes it necessary to set a large bandwidth in the phase-locked loop (the bandwidth is not less than 10 Hz during the experiments), otherwise, the closed-loop circuit will fail to track the frequency of the charge sensor. The bandwidth of the phase-locked loop determines the corner frequency of the error signal in the phase-locked loop. The fluctuations on a faster time scale than the transfer function will be filtered out. Therefore, a large bandwidth will also degrade the frequency stability, limiting the resolution of the charge sensor. The trade-off between frequency stability and frequency tracking ability in the phase-locked loop hinders further performance improvement in our sensing scheme. A more suitable closed-loop circuit or an optimized phase-locked loop will help further demonstrate the sensing capabilities of our sensing scheme.

## Conclusion

In conclusion, we have proposed and implemented a scheme that allows a significant enhancement in measurement sensitivity. The scheme tracks the frequency of the top saddle-node bifurcation in the parametric isolated branch to reflect the external perturbation. The frequency of the top bifurcation undergoes a dramatic decrease before its annihilation, which significantly boosts the sensing sensitivity. The proposed sensing scheme enables the detection of external perturbations that affect the linear stiffness, such as inertial force and electrostatic force. We implemented a closed-loop charge detection to validate the proposed sensing scheme. Charge sensitivity of up to 39.5 $\text{ppm}\cdot {f\text{C}}^{-1}$ was achieved. The charge sensor was demonstrated with a bias instability of 7.34 electrons and a noise floor of 8.41 e/${\text{Hz}}^{-1/2}$. The double hysteresis phenomena offer intriguing opportunities for utilizing a single resonator to design high-performance resonant force sensors. This capability allows for high measurement sensitivity, small device footprint, and simplified design for resonator and circuit, thus inspiring rapid and precise sensing applications.

## Materials and methods

### Experimental setup

The device is operated at room temperature. The parametric pump and direct external drive signals are generated by a lock-in amplifier (Zurich Instruments HF2LI). The DC bias is generated by a low noise power supply (Keysight E3649A). The DC voltage ${V}_{e}$ is generated by a high-voltage power supply (Keysight 2400). The vibration motion is detected in the form of a motional current, which is amplified utilizing a transimpedance amplifier (TIA) and demodulated by the lock-in amplifier. During open-loop experiments, the parametric pump and external drive are simultaneously applied on the driving electrode through a built-in signal adder in the lock-in amplifier. During closed-loop experiments, the direct external drive is applied on an additional stimulus input electrode D to break the symmetry of the degenerated phase states.

### Stability analysis

In order to unveil the mechanism for the double hysteresis and determine the stabilities of the theoretical solutions, we first recast Eq. (2) and use the averaging method for deriving the differential equations of slow flow in terms of $p={acos}(\psi )$ and $q={asin}(\psi )$ where $a$ and $\psi$ are the slowly-varying polar coordinate representing the amplitude and the phase of the transverse motion $u$. The slow time equation for $p$ and $q$ can be obtained as

3 $$\dot{p}=-\frac{1}{2\omega }\left(\frac{\omega }{Q}p+\left(\delta +\frac{\lambda }{2}\right)q+\frac{3}{4}{\kappa }^{{eff}}\left({p}^{2}+{q}^{2}\right)q+\frac{\omega \eta }{4}\left({p}^{2}+{q}^{2}\right)p-{hsin}{\varphi }_{0}\right)$$

4 $$\dot{q}=-\frac{1}{2\omega }\left(\frac{\omega }{Q}q+\left(-\delta +\frac{\lambda }{2}\right)p-\frac{3}{4}{\kappa }^{{eff}}\left({p}^{2}+{q}^{2}\right)p+\frac{\omega \eta }{4}\left({p}^{2}+{q}^{2}\right)q+{hcos}{\varphi }_{0}\right)$$

The detailed process for derivation of the phase portrait equations is presented in Supplementary Section 2. The phase portrait is then determined by numerically integrating Eq. (3) and Eq. (4) utilizing a customized ode45 program. The phase portraits show the evolution of stable attractors and unstable saddle points. The square boxes represent the stable solutions, and the round boxes represent the unstable solutions.

**Fig. 7:**
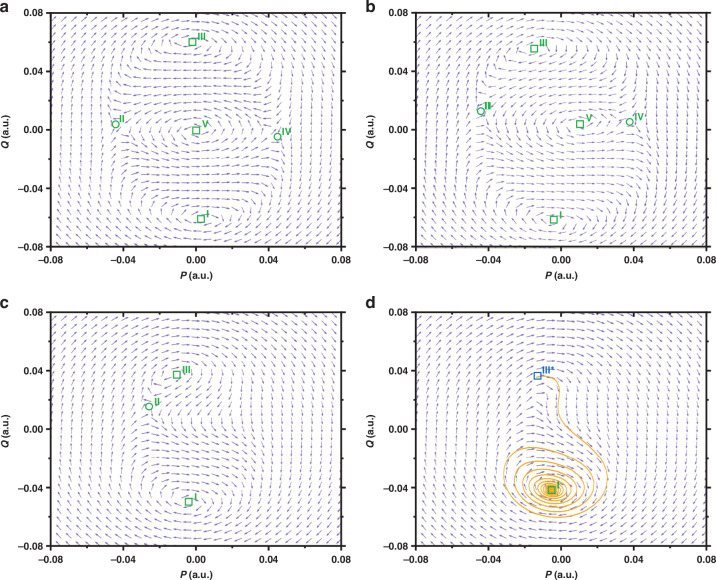
The phase portraits of the nonlinear parametric resonator in a pair of orthogonal slow variables *p* and *q.* **a** shows the phase portrait of the system before symmetry breaking happens. In this case, the solutions are degenerate in amplitude. The square boxes represent the stable solutions, and the round boxes represent the unstable solutions. **b**–**d** Show the phase portraits after the direct external driving voltage is injected into the system when driving frequency $\varOmega$ is equal to $0.9993{\omega }_{0}$,$\,0.9997{\omega }_{0}$ and $0.9999{\omega }_{0}$, respectively. In these cases, the symmetry breaking happens and the degeneracy of the solutions is broken. The orange solid line in **d** represents the stable manifold for oscillation state switching during upward frequency sweep. The parameters used to map the phase portraits are the same as those in Fig. 3a

Figure 7a shows the phase portrait in the absence of the direct external drive. The parameters used to map the phase portrait hereafter are the same as those in Fig. 3a, unless otherwise specified. The frequency of the parametric pump is set to twice the $0.9993{\omega }_{0}$ (in domain (1) shown in Fig. 3b). The nontrivial stable solutions I and III are degenerate in amplitude. Similarly, two saddle points II and IV are also degenerate. Since the nontrivial stable solutions I and III have a larger amplitude compared to the two saddle points II and IV, the nontrivial stable solutions I and III correspond to the stable upper branches before symmetry breaking happens, while the two saddle points II and IV correspond to the unstable lower branches before symmetry breaking happens.

Next, in order to determine the stabilities of the theoretical solutions after symmetry breaking happens, we plot the phase portraits of the system in the presence of the direct external drive, as shown in Figs. 7b–d. When the frequency of the parametric pump is set to twice the $0.9993{\omega }_{0}$ (in domain (1) shown in Fig. 3b). Obviously, the degeneracies of both the nontrivial stable solutions (I and III) and saddle points (II and IV) are broken, which indicates that symmetry breaking happens. The nontrivial stable solution I has a larger amplitude compared to III which implies that the stable solution I corresponds to the upper main branch i (stable) and the stable solution III corresponds to the upper parametric isolated branch iii (stable). Similarly, the saddle point II has a larger amplitude compared to the saddle point IV which implies that the saddle point II corresponds to the lower parametric isolated branch ii (unstable) and the saddle point IV corresponds to the lower main branch iv (unstable). Based on the above results, the stabilities of the theoretical solutions in the symmetry breaking state are determined.

When the driving frequency is increased from $0.9993{\omega }_{0}$ to $0.9997{\omega }_{0}$ (from domain (1) to domain (2)), the stable solution V (corresponding to the branch v) and the saddle point IV (corresponding to the lower main branch iv) disappear in the phase portrait. Further increasing the driving frequency from $0.9997{\omega }_{0}$ to $0.9999{\omega }_{0}$ (from domain (2) to domain (3)) will result in the annihilation of the stable solution III (corresponding to the branch iii) and the saddle point II (corresponding to the lower parametric isolated branch ii). We take the state of stable solution III in Fig. 7c as the initial state and perform numerical integration under the corresponding system parameters in Fig. 7d. As shown in Fig. 7d, this initial state is donated by III*, and the orange manifold demonstrates the oscillation state switching during the upward frequency sweeping from $0.9997{\omega }_{0}$ to $0.9999{\omega }_{0}$. The oscillation state switches from the isolated branch iii to the main branch i, resulting in double hysteresis in the frequency responses.

### Theoretical validation of sensitivity enhancement in charge sensing

To further interpret this sensitivity enhancement scheme in charge sensing, we derive an analytical expression for the size of the parametric isolated branches utilizing the averaging method:

5 $$-\frac{\eta {a}_{I}^{3}}{2}\left(1+\frac{\left(3\beta -10{\alpha }^{2}/3\right){a}_{I}^{2}}{8}\right)+\left(\lambda +\frac{2}{Q}\left(1+\frac{\left(3\beta -10{\alpha }^{2}/3\right){a}_{I}^{2}}{8}\right)\right){a}_{I}=2h$$

where ${a}_{I}$ is the amplitude of the saddle-node bifurcations in the parametric isolated branches. The bifurcation frequencies thus can be obtained by substituting the calculated amplitude into the backbone line formula $\omega =1+\left(3\beta -10{\alpha }^{2}/3\right)/8$. The detailed derivation process is presented in Supplementary Section 2.

**Fig. 8:**
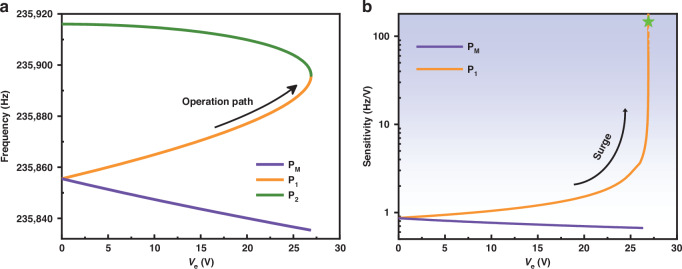
Theoretical validation of the sensitivity enhancement. **a** The theoretical relationship between the frequencies of the bifurcation points and the perturbation DC voltage. **b** Theoretical measurement sensitivity

Figure 8a shows the theoretical relationship between the bifurcation frequencies and the perturbation DC voltage ${V}_{e}$ according to Eq. (5). As ${V}_{e}$ increases, bifurcation points ${P}_{1}$ and ${P}_{2}$ gradually approach with an increasing rate and finally annihilate at a frequency of 235895.85 Hz. In comparison, the frequency of the top saddle-node bifurcation ${P}_{M}$ in the main branch decreases at an almost constant rate. Measurement sensitivity is then calculated, as depicted in Fig. 8b. The sensitivity of the bifurcation ${P}_{1}$ surges from 0.940 Hz/V at ${V}_{e}=0\text{V}$ to 146 Hz/V (35.4 $\text{ppm}\cdot {f\text{C}}^{-1}$) at ${V}_{e}\text{=}26.9\text{V}$ (shown in green pentagram), far exceeding the sensitivity of the bifurcation ${P}_{M}$.

## Supplementary information

Suplementary
