# Supplementary material for: Sensitivity enhancement of nonlinear micromechanical sensors using parametric symmetry breaking
Source: Microsyst Nanoeng. 2024 Oct 29;10:158. doi: 10.1038/s41378-024-00784-4 (PMC11519502; doi:10.1038/s41378-024-00784-4)
Supplement: Supplementary file 1 — Suplementary [file 41378_2024_784_MOESM1_ESM.docx]

# SUPPLEMENTARY INFORMATION

# Sensitivity enhancement of nonlinear micromechanical sensors using parametric symmetry breaking

Yutao Xu^1^, Qiqi Yang^1^, Jiahao Song^1^ and Xueyong Wei^1, 2,^ *

^1^State Key Laboratory for Manufacturing Systems Engineering, Xi’an Jiaotong University, Xi’an, 710049, China

^2^School of Instrument Science and Technology, Xi’an Jiaotong University, Xi’an, 710049, China

^*^ Correspondence: Xueyong Wei ([seanwei@mail.xjtu.edu.cn](mailto:seanwei@mail.xjtu.edu.cn))

**Supplementary Section 1. Mathematical modelling and perturbation analysis**

- 1. Mathematical modelling


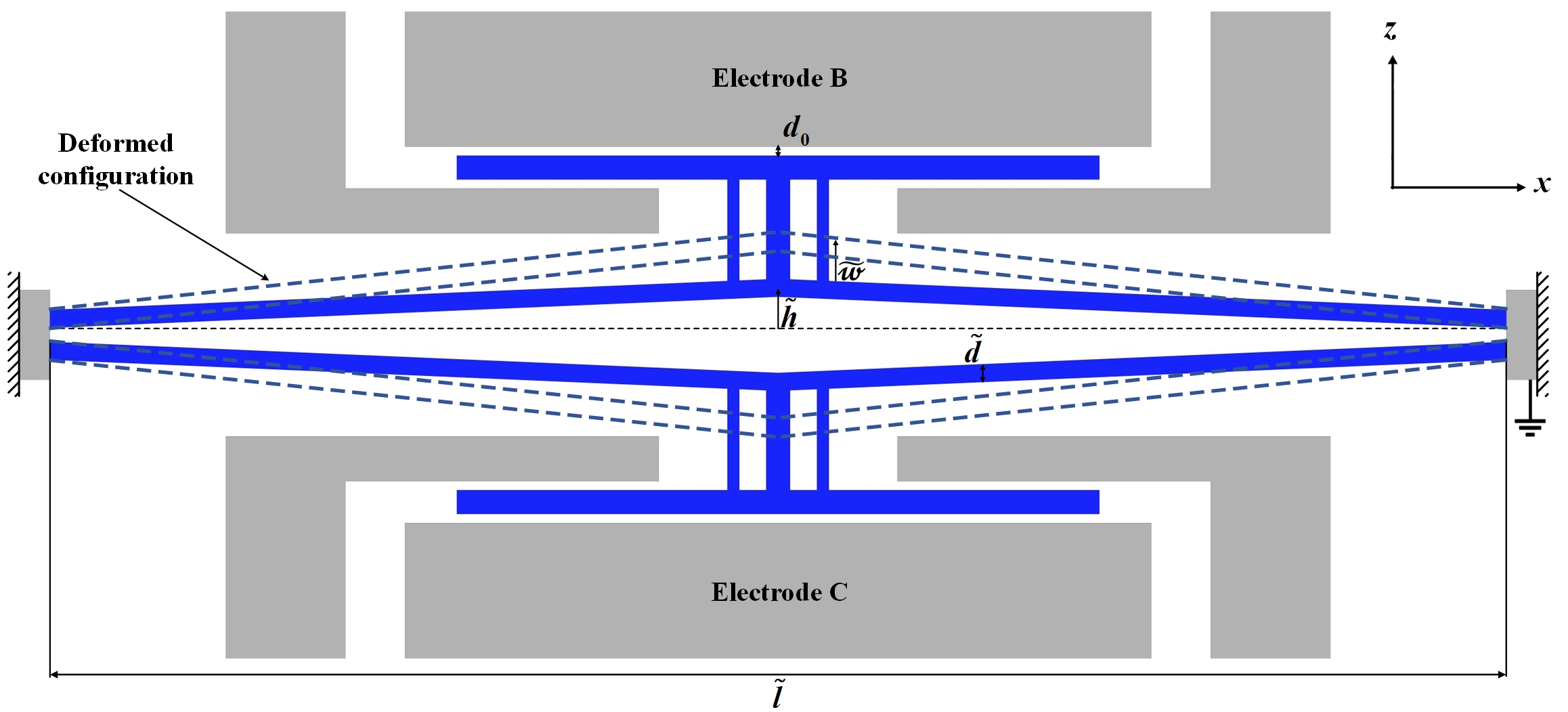


**Supplementary Figure S1.** Schematic of the parametrically excited diamond-shaped resonator considered for analysis.

The diamond-shaped MEMS resonator consists of two identical arch beams of length $\tilde{l}$=487.9 μm, width $\tilde{d}$=6.0 μm, thickness $\tilde{b}$=25.0 μm, gap width $d_{0}$=3 μm, and arch height $\tilde{h}$=5.964 μm, as shown in Fig. S1. The equation governing the dynamic behaviors of the shallow V-shaped micro resonator is derived based on the device structures. The actual profile of the microbeam is

$\mathcal{w}_{0}\left( x \right)=\left\{ \begin{aligned} \frac{2\tilde{h}}{\tilde{l}}x, &0\leq x<\frac{1}{2} \\ -\frac{2\tilde{h}}{\tilde{l}}x+\frac{2\tilde{h}}{\tilde{l}}, &\frac{1}{2}\leq x\leq1 \end{aligned} \right.$ (S1)

where $\tilde{h}$ and $\tilde{l}$ represent the initial rise at the mid-point and the length of the microbeam, respectively.

We follow the Euler-Bernoulli beam model and Kevin-Voigt model while accounting for the geometric, electrostatic, and damping nonlinearities to formulate the mathematical model of the proposed parametrically driven MEMS sensor^1,2^. The equation of motion governing the transverse deflection $\tilde{\mathcal{w}}$ of the MEMS sensor with both parametric drive and direct external drive can be described as

$\left( \rho A+m_{c}\delta\left( \tilde{x}-\frac{\tilde{l}}{2} \right) \right)\frac{\partial^{2}\tilde{\mathcal{w}}}{\partial\tilde{t}^{2}}+EI\frac{\partial^{4}\tilde{\mathcal{w}}}{\partial\tilde{x}^{4}}+\tilde{C}\frac{\partial\tilde{\mathcal{w}}}{\partial\tilde{t}}+\xi I\frac{\partial^{5}\tilde{\mathcal{w}}}{\partial\tilde{t}\partial\tilde{x}^{4}}-\frac{EA}{2\tilde{l}}\left[ \frac{\partial^{2}\tilde{\mathcal{w}}}{\partial\tilde{x}^{2}}+\frac{\partial^{2}{\tilde{\mathcal{w}}}_{0}}{\partial\tilde{x}^{2}} \right]\int_{0}^{\tilde{l}} \left[ \left( \frac{\partial\tilde{\mathcal{w}}}{\partial\tilde{x}} \right)^{2}+2\left( \frac{\partial{\tilde{\mathcal{w}}}_{0}}{\partial\tilde{x}}\frac{\partial\tilde{\mathcal{w}}}{\partial\tilde{x}} \right) \right]d\tilde{x}-\frac{\xi A}{2\tilde{l}}\left[ \frac{\partial^{2}\tilde{\mathcal{w}}}{\partial\tilde{x}^{2}}+\frac{\partial^{2}{\tilde{\mathcal{w}}}_{0}}{\partial\tilde{x}^{2}} \right]\int_{0}^{\tilde{l}} \left[ 2\left( \frac{\partial^{2}\tilde{\mathcal{w}}}{\partial\tilde{t}\partial\tilde{x}}\frac{\partial\tilde{\mathcal{w}}}{\partial\tilde{x}} \right)+2\left( \frac{\partial{\tilde{\mathcal{w}}}_{0}}{\partial\tilde{x}}\frac{\partial^{2}\tilde{\mathcal{w}}}{\partial\tilde{t}\partial\tilde{x}} \right) \right]d\tilde{x}=\tilde{\Lambda}\left( \tilde{\mathcal{w}},\tilde{\Omega}\tilde{t} \right)\delta\left( \tilde{x}-\frac{\tilde{l}}{2} \right)+\tilde{H}\left( \tilde{\Omega}\tilde{t} \right)\delta\left( \tilde{x}-\frac{\tilde{l}}{2} \right)$ (S2)

subject to the following boundary conditions:

$\tilde{\mathcal{w}}\left( 0,\tilde{t} \right)=\tilde{\mathcal{w}}\left( \tilde{l},\tilde{t} \right)=0$ (S3)

$\frac{\partial\tilde{\mathcal{w}}\left( 0,\tilde{t} \right)}{\partial\tilde{x}}=\frac{\partial\tilde{\mathcal{w}}\left( \tilde{l},\tilde{t} \right)}{\partial\tilde{x}}=0$ (S4)

where ${\tilde{\mathcal{w}}}_{0}$ represents the microbeam profile, $\rho$ is the density, $A$ is the area of the cross-section of the microbeam, $m_{c}$ is the concentrated mass in the middle, $\tilde{x}$ is the position along the microbeam length, $\tilde{l}$ represents the length of the microbeam, $E$ is the Young’s modulus, $\tilde{C}$ is the linear damping per unit length, $\xi$ is the viscous damping coefficient, $I$ is the moment of inertia of the cross-section, $\tilde{\Lambda}$ is the strength of the parametric pump, $\tilde{H}$ is the strength of direct external drive, $\tilde{t}$ is the time, $\tilde{\Omega}$ is the driving frequency, and $\delta$ is the Dirac function. Rescaling the spatial variables by $\tilde{l}$ ($\mathcal{w=}{\tilde{\mathcal{w}}}/\tilde{l}$, $\mathcal{w}_{0}={{\tilde{\mathcal{w}}}_{0}}/\tilde{l}$, $x=\tilde{x}/\tilde{l}$) and time by $t=\tilde{t}\sqrt{\frac{EI}{\rho A\tilde{l}^{4}}}$, the non-dimensional governing equation can be obtained as

$\left( 1+\bar{m}\delta\left( x-\frac{1}{2} \right) \right)\frac{\partial^{2}\mathcal{w}}{\partial t^{2}}+\frac{\partial^{4}\mathcal{w}}{\partial x^{4}}+C\frac{\partial\mathcal{w}}{\partial t}-\alpha_{1}\left( \frac{\partial^{2}\mathcal{w}}{\partial x^{2}}+\frac{\partial^{2}\mathcal{w}_{0}}{\partial x^{2}} \right)\int_{0}^{1} \left( \left( \frac{\partial\mathcal{w}}{\partial x} \right)^{2}+2\left( \frac{\partial\mathcal{w}_{0}}{\partial x}\frac{\partial\mathcal{w}}{\partial x} \right)- \right)dx-\alpha_{2}\left( \frac{\partial^{2}\mathcal{w}}{\partial x^{2}}+\frac{\partial^{2}\mathcal{w}_{0}}{\partial x^{2}} \right)\int_{0}^{1} \left( 2\left( \frac{\partial^{2}\mathcal{w}}{\partial t\partial x}\frac{\partial\mathcal{w}}{\partial x} \right)+2\left( \frac{\partial\mathcal{w}_{0}}{\partial x}\frac{\partial^{2}\mathcal{w}}{\partial t\partial x} \right) \right)dx=\Lambda\left( \mathcal{w,}\Omega t \right)\delta\left( x-\frac{1}{2} \right)+H\left( \Omega t \right)\delta\left( x-\frac{1}{2} \right)$ (S5)

subject to

$\mathcal{w}\left( 0,t \right)\mathcal{=w}\left( 1,t \right)=0$ (S6)

$$\begin{aligned} \frac{\partial\mathcal{w}\left( 0,t \right)}{\partial x}=\frac{\partial\mathcal{w}\left( 1,t \right)}{\partial x}=0\#\left( S7 \right) \end{aligned}$$

where $\alpha_{1}=6\left( \tilde{l}/\tilde{d} \right)^{2}$ (represents the aspect ratio parameter), $\alpha_{2}={\sqrt{3}\xi}/{\sqrt{\rho E}\tilde{d}}$, $\bar{m}={m_{c}}/{\rho A\tilde{l}}$, $C={\tilde{C}\tilde{l}^{2}}/\sqrt{\rho AEI}$, and $\Omega=\tilde{\Omega}\sqrt{\frac{\rho A\tilde{l}^{4}}{EI}}$. $\Lambda\left( \mathcal{w,}\Omega t \right)$ represents the dimensionless parametric excitation, and $H\left( \Omega t \right)$ represents the dimensionless direct external drive. Viscous damping terms other than quadratic order are ignored for simplicity.

To examine the full dynamics of the parametrically driven system under the injection of a direct external drive, we employ the Galerkin method to derive a reduced-order model. Thus, the transverse deflection is expanded as follows:

$\mathcal{w}\left( x,t \right)=\sum_{i=1}^{N} u_{i}\left( t \right)\phi_{i}\left( x \right)$ (S8)

where $u_{i}(t)$ is the $i^{th}$ order time-varying generalized coordinate for the transverse motion, and $\phi_{i}(x)$ is the corresponding normalized non-dimensional undamped mode shape for a linear clamped-clamped beam with a concentrated mass in the middle. The mode shape satisfies^3^:

$$\begin{aligned} \frac{\partial^{4}\phi_{i}\left( x \right)}{\partial x^{4}}=p_{i}^{2}\left( 1+\bar{m}\delta\left( x-\frac{1}{2} \right) \right)\phi_{i}\left( x \right)\#\left( S9 \right) \end{aligned}$$

where $p_{i}$ is equal to the $i^{th}$ order non-dimensional natural frequency $\omega_{i}$.

Eqs. (S6), (S7) and (S9) define an eigenvalue problem, the solution of which can be obtained by Laplace transform. Let the transformed quantities be denoted by $\bar{\phi}$.Then

$$\begin{aligned} \bar{\phi}_{i}={\phi_{i}}^{''}\left( 0 \right)\frac{s}{s^{4}-{k_{i}}^{4}}+{\phi_{i}}^{'''}\left( 0 \right)\frac{1}{s^{4}-{k_{i}}^{4}}+\bar{m}p_{i}^{2}\phi_{i}\left( \frac{1}{2} \right)\frac{e^{-\frac{1}{2}s}}{s^{4}-{k_{i}}^{4}}\#\left( S10 \right) \end{aligned}$$

where the prime symbol denotes differentiation with respect to the non-dimensional axial $x$. $k_{i}$ is equal to the square root of the $i^{th}$ order non-dimensional natural frequency $\omega_{i}$ ($k_{i}^{4}=p_{i}^{2}$).

Hence

$\phi_{i}\left( x \right)=\frac{{\phi_{i}}^{''}\left( 0 \right)\left( \cosh\left( k_{i}x \right)-\cos\left( k_{i}x \right) \right)}{2k_{i}^{2}}+\frac{{\phi_{i}}^{'''}\left( 0 \right)\left( \sinh\left( k_{i}x \right)-\sin\left( k_{i}x \right) \right)}{2k_{i}^{3}}+\frac{\bar{m}p_{i}^{2}\phi_{i}\left( \frac{1}{2} \right)}{2k_{i}^{3}}U\left( x-\frac{1}{2} \right)\left[ \sinh\left[ k_{i}\left( x-\frac{1}{2} \right) \right]-sin\left[ k_{i}\left( x-\frac{1}{2} \right) \right] \right]$ (S11)

where $U\left( x-\frac{1}{2} \right)$ is a Heaviside function at $x=\frac{1}{2}$. The remaining two unknown $\phi_{i}^{''}\left( 0 \right)$ and ${\phi_{i}}^{'''}\left( 0 \right)$ in the mode shape function can be determined from the remaining two boundary conditions of Eq. (S6) and (S7). The results obtained after some algebraic manipulations are

${\phi_{i}}^{''}\left( 0 \right)=-\frac{\bar{m}p_{i}^{2}\phi_{i}\left( \frac{1}{2} \right)}{k_{i}\left[ cos\left( k_{i} \right)cosh\left( k_{i} \right)-1 \right]}\left[ cos\left( \frac{1}{2}k_{i} \right)-cosh\left( \frac{1}{2}k_{i} \right) \right]\left[ sinh\left( \frac{1}{2}k_{i} \right)cos\left( \frac{1}{2}k_{i} \right)-sin\left( \frac{1}{2}k_{i} \right)cosh\left( \frac{1}{2}k_{i} \right) \right]$ (S12)

${\phi_{i}}^{'''}\left( 0 \right)=-\frac{2\bar{m}p_{i}^{2}\phi_{i}\left( \frac{1}{2} \right)}{{sinh}^{2}\left( k_{i} \right)+2cos\left( k_{i} \right)cosh\left( k_{i} \right)-{cosh}^{2}(k_{i})-1}\left[ \cos\left( \frac{1}{2}k_{i} \right)\left[ sinh\left( \frac{1}{2}k_{i} \right)sin\left( \frac{1}{2}k_{i} \right)-1 \right]+cosh\left[ -sinh\left( \frac{1}{2}k_{i} \right)sin\left( \frac{1}{2}k_{i} \right)+{cos}^{2}\left( \frac{1}{2}k_{i} \right)-1 \right]+{cosh}^{2}\left( \frac{1}{2}k_{i} \right)cos\left( \frac{1}{2}k_{i} \right) \right]$ (S13)

Substituting Eqs. (S12) and (S13) into Eq. (S11), we can obtain the eigenfunction as

$\phi_{i}\left( x \right)=\frac{\bar{m}p_{i}^{2}\phi_{i}\left( \frac{1}{2} \right)}{4k_{i}^{3}\left[ cos\left( k_{i} \right)\cosh\left( k_{i} \right)-1 \right]}\left[ \left[ 2U\left( x-\frac{1}{2} \right)+1 \right]\left[ sin\left[ k_{i}\left( x-\frac{1}{2} \right) \right]-sinh\left[ k_{i}\left( x-\frac{1}{2} \right) \right] \right]+\left[ U\left( x-\frac{1}{2} \right)-1 \right]\left[ sinh\left[ k_{i}\left( x-\frac{1}{2} \right) \right]cos\left( k_{i} \right)-sin\left[ k_{i}\left( x-\frac{1}{2} \right) \right]cosh\left( k_{i} \right) \right]+U\left( x-\frac{1}{2} \right)\left[ sinh\left[ k_{i}\left( x-\frac{3}{2} \right) \right]cos\left( k_{i} \right)-sin\left[ k_{i}\left( x-\frac{3}{2} \right) \right]cosh\left( k_{i} \right) \right]+\left[ sinh\left( \frac{1}{2}k_{i} \right)cos\left( k_{i}x \right)-sin\left( \frac{1}{2}k_{i} \right)cosh\left( k_{i}x \right) \right]+\left[ sinh\left( k_{i}x \right)cos\left( \frac{1}{2}k_{i} \right)-sin\left( k_{i}x \right)cosh\left( \frac{1}{2}k_{i} \right) \right]-\left[ sinh\left[ k_{i}\left( x-1 \right) \right]cos\left( \frac{1}{2}k_{i} \right)-sin\left[ k_{i}\left( x-1 \right) \right]cosh\left( \frac{1}{2}k_{i} \right) \right]+\left[ sinh\left( \frac{1}{2}k_{i} \right)cos\left[ k_{i}\left( x-1 \right) \right]-sin\left( \frac{1}{2}k_{i} \right)cosh\left[ k_{i}\left( x-1 \right) \right] \right]-\left[ sinh\left( k_{i}x \right)cos\left[ k_{i}\left( x-\frac{1}{2} \right) \right]-sin\left( k_{i}x \right)cosh\left[ k_{i}\left( x-\frac{1}{2} \right) \right] \right] \right]$ (S14)

The eigenvalue $k_{i}$ is equal to the square root of the $i^{th}$ order non-dimensional natural frequency. In order to obtain the infinite set of eigenvalues $k_{i}$ and the expression of the transverse deflection $\mathcal{w(}x)$, we let $x=\frac{1}{2}$ in Eq. (S14) and get

$k_{i}cos\left( k_{i} \right)cosh\left( k_{i} \right)+\bar{m}p_{i}^{2}\left[ \left[ cos\left( \frac{1}{2}k_{i} \right)-cosh\left( \frac{1}{2}k_{i} \right) \right]\left[ sinh\left( \frac{1}{2}k_{i} \right)-cos\left( \frac{1}{2}k_{i} \right) \right]-sin\left( \frac{1}{2}k_{i} \right)cosh\left( \frac{1}{2}k_{i} \right) \right]=1$ (S15)

The eigenfunctions of mode shape can thus be derived by substituting the eigenvalues $k_{i}$ into Eq. (S14). We perform a Galerkin projection to obtain the reduced-order nonlinear ordinary differential equation. Eq. (S8) is substituted into Eq. (S5). The outcome is then multiplied by the mode shape $\phi_{j}$ and is integrated over the beam domain from 0 to 1^4^:

$\int_{0}^{1} \phi_{j}\left( x \right)\varepsilon\left( x \right)\left( \sum_{1}^{N} \phi_{i}\left( x \right)\ddot{u}_{i}\left( t \right) \right)dx+\int_{0}^{1} \phi_{j}\left( x \right)\left( \sum_{i=1}^{N} \phi_{i}^{''''}\left( x \right)u_{i}\left( t \right) \right)dx+c\int_{0}^{1} \phi_{j}\left( x \right)\left( \sum_{i=1}^{N} \phi_{i}\left( x \right)\dot{u}_{i}\left( t \right) \right)dx-2\alpha_{1}\sum_{i,j,k=1}^{N} u_{i}u_{k}\int_{0}^{1} \phi_{j}\left( x \right)\phi_{i}^{''}\left( x \right)dx\int_{0}^{1} {\frac{\partial\mathcal{w}_{0}\left( x \right)}{\partial x}\phi}_{k}^{'}\left( x \right)dx-\alpha_{1}\sum_{i,j,k,l=1}^{N} u_{i}u_{k}u_{l}\int_{0}^{1} \phi_{j}\left( x \right)\phi_{i}^{''}\left( x \right)dx\int_{0}^{1} \phi_{k}^{'}\left( x \right)\phi_{l}^{'}\left( x \right)dx-2\alpha_{2}\sum_{i,j,k=1}^{N} u_{i}u_{k}^{'}\int_{0}^{1} \phi_{j}\left( x \right)\phi_{i}^{''}\left( x \right)dx\int_{0}^{1} {\frac{\partial\mathcal{w}_{0}\left( x \right)}{\partial x}\phi}_{k}^{'}\left( x \right)dx-2\alpha_{2}\sum_{i,j,k,l=1}^{N} u_{i}u_{k}u_{l}^{'}\int_{0}^{1} \phi_{j}\left( x \right)\phi_{i}^{''}\left( x \right)dx\int_{0}^{1} \phi_{k}^{'}\left( x \right)\phi_{l}^{'}\left( x \right)dx=\Lambda\left( \mathcal{w},\Omega t \right)\delta\left( x-\frac{1}{2} \right)$ (S16)

where $\varepsilon\left( x \right)=1+\bar{m}\delta\left( x-\frac{1}{2} \right)$. It was proved that when the microbeam is driven in the primary resonance near the first natural frequency, the first mode is dominant, and the other modes can be neglected. A dynamic model for the shallow arch micromechanical beam can then be developed involving only the dominant first mode by letting N=1 in Eq. (S16):

$\ddot{u}_{1}\left( t \right)\int_{0}^{1} \varepsilon\left( x \right)dx+{\omega_{1}^{2}u}_{1}\left( t \right)+c\dot{u}_{1}\left( t \right)-2\alpha_{1}u_{1}^{2}\int_{0}^{1} \phi_{1}\left( x \right)\phi_{1}^{''}\left( x \right)dx\int_{0}^{1} {\frac{\partial\mathcal{w}_{0}\left( x \right)}{\partial x}\phi}_{1}^{'}\left( x \right)dx-\alpha_{1}u_{1}^{3}\int_{0}^{1} \phi_{1}\left( x \right)\phi_{1}^{''}\left( x \right)dx\int_{0}^{1} \left( \phi_{1}^{'}\left( x \right) \right)^{2}dx-2\alpha_{2}u_{1}\dot{u}_{1}\int_{0}^{1} \phi_{1}\left( x \right)\phi_{1}^{''}\left( x \right)dx\int_{0}^{1} {\frac{\partial\mathcal{w}_{0}\left( x \right)}{\partial x}\phi}_{1}^{'}\left( x \right)dx-2\alpha_{2}u_{1}^{2}\dot{u}_{1}\int_{0}^{1} \phi_{1}\left( x \right)\phi_{1}^{''}\left( x \right)dx\int_{0}^{1} \left( \phi_{1}^{'}\left( x \right) \right)^{2}dx=\Lambda\left( \mathcal{w},\Omega t \right)\delta\left( x-\frac{1}{2} \right)$ (S17)

where $\phi_{i}^{''''}\left( x \right)$ is replaced with $\omega_{1}^{2}\phi_{1}\left( x \right)$. The orthonormality of the mode shapes^4^ is used to simplify the governing equation. It is worth noting that, due to the axisymmetric characteristics of both the first mode and the initial shape, we have

$\int_{0}^{1} \frac{\partial\mathcal{w}_{0}\left( x \right)}{\partial x}\phi_{1}^{'}\left( x \right)dx=\frac{2\tilde{h}}{\tilde{l}}\int_{0}^{\frac{1}{2}} \phi_{1}^{'}\left( x \right)dx$ (S18)

$\int_{0}^{1} \frac{\partial\mathcal{w}_{0}\left( x \right)}{\partial x}\left( \phi_{1}^{'}\left( x \right) \right)^{3}dx=\frac{2\tilde{h}}{\tilde{l}}\int_{0}^{\frac{1}{2}} \left( \phi_{1}^{'}\left( x \right) \right)^{3}dx$ (S19)

Thus, Eq. (S17) can be further simplified as

$M\frac{d^{2}u_{1}(t)}{dt^{2}}+C\frac{du_{1}(t)}{dt}+{\omega_{1}^{2}u}_{1}\left( t \right)+K_{2}u_{1}^{2}+K_{3}u_{1}^{3}+H_{3}u_{1}^{2}\dot{u}_{1}=\Lambda_{e}+H_{e}$ (S20)

and the coefficients are

$$M=\int_{0}^{1} \left( 1+\bar{m}\delta\left( x-\frac{1}{2} \right) \right)dx$$

$$K_{2}=-\frac{4\tilde{h}}{\tilde{l}}\alpha_{1}\int_{0}^{1} \phi_{1}\left( x \right)\phi_{1}^{''}\left( x \right)dx\int_{0}^{\frac{1}{2}} \phi_{1}^{'}\left( x \right)dx$$

$$K_{3}=-\alpha_{1}\int_{0}^{1} \phi_{1}\left( x \right)\phi_{1}^{''}\left( x \right)dx\int_{0}^{1} \left( \phi_{1}^{'}\left( x \right) \right)^{2}dx$$

$$H_{3}=-2\alpha_{2}\int_{0}^{1} \phi_{1}\left( x \right)\phi_{1}^{''}\left( x \right)dx\int_{0}^{1} \left( \phi_{1}^{'}\left( x \right) \right)^{2}dx$$

where only the third-order nonlinear damping is retained for simplicity. The governing equation of motion can be rewritten by considering the dimensionless as follows:

$\ddot{u}+\frac{1}{Q}\dot{u}+\left( 1-\lambda\cos\left( 2\omega\tau\right) \right)u+\alpha u^{2}+\beta u^{3}+\eta u^{2}\dot{u}=hcos(\omega\tau+\varphi_{0})$ (S21)

where the time and displacement are refined according to $\tau=\omega_{0}t$, and $\omega_{0}=\sqrt{{\omega_{1}^{2}}/m}$. The dot overhead symbol denotes differentiation with respect to the dimensionless time $\tau$. Dimensionless parameters are defined as $u={\frac{\tilde{l}}{d_{0}}u}_{1}$, $Q^{-1}=\frac{C}{M\omega_{0}\tilde{l}}$, $\alpha=\frac{K_{2}}{M\omega_{0}^{2}}\frac{d_{0}}{\tilde{l}}$, $\beta=\frac{K_{3}}{M\omega_{0}^{2}}\left( \frac{d_{0}}{\tilde{l}} \right)^{2}$, $\eta=\frac{H_{3}}{M\omega_{0}}\left( \frac{d_{0}}{\tilde{l}} \right)^{2}$, $\omega=\frac{\Omega}{\omega_{0}}$, $\lambda=\frac{\tilde{\lambda}}{M\omega_{0}^{2}}\frac{\tilde{l}}{d_{0}}$, $h=\frac{\tilde{h}}{M\omega_{0}^{2}}\frac{\tilde{l}}{d_{0}}$. $\varphi_{0}$ is the phase difference (PD) between the parametric pump and direct external drive. The strength of parametric pump $\tilde{\lambda}$ and direct external drive $\tilde{h}$ can be obtained through Taylor expansion as

$\tilde{\lambda}=\frac{2\varepsilon_{0}sV_{dc1}V_{pump}}{d_{0}^{3}}$ (S22)

$\tilde{h}=\frac{\varepsilon_{0}sV_{dc2}V_{dir}}{d_{0}^{2}}$ (S23)

where $\varepsilon_{0}$ is the permittivity of vacuum, *S* is the area of the excitation electrode plate, $V_{dci}$ $(i=1,2)$ is the magnitude of DC polarization voltage, $V_{dir}$ is the AC voltage for the direct external drive, and $V_{pump}$ is the AC voltage for parametric pump.

1.2 Perturbation analysis

To study the response characteristics of our parametrically driven system before and after being perturbated by the direct external drive voltage $V_{dir}$, we implement the method of multiple scales (MMS) to obtain an approximate solution. The first and second approximations of the MMS are applied to Eq. (S21) to obtain the approximate solution.

We apply the MMS by introducing

${u=u}_{0}\left( T_{0},T_{1},T_{2} \right)+\epsilon u_{1}\left( T_{0},T_{1},T_{2} \right)+\epsilon^{2}u_{2}\left( T_{0},T_{1},T_{2} \right)$ (S24)

where $\epsilon$ is the infinitesimal, $T_{0}=\tau$ is the fast time scale, $T_{1}=\epsilon T_{0}$ is the first-order slow time scale, and $T_{2}=\epsilon^{2}T_{0}$ represents the second-order slow time scale. Time derivatives are transformed into

$\begin{matrix} D=D_{0}+\epsilon D_{1}+\epsilon^{2}D_{2} \\ D^{2}={D_{0}}^{2}+2\epsilon D_{0}D_{1}+\epsilon^{2}\left( {{2D}_{0}D}_{2}+D_{1}^{2} \right) \end{matrix}$ (S25)

Substituting Eqs. (S24) and (S25) into Eq. (S21) and equating the coefficients of the same order of $\epsilon$ on both sides, we get $\epsilon^{0}$, $\epsilon^{1}$, and $\epsilon^{2}$ order equations as

$\epsilon^{0}$ order equation:

${D_{0}}^{2}u_{0}+u_{0}=0$ (S26)

$\epsilon^{1}$ order equation:

${D_{0}}^{2}u_{1}+u_{1}=-\alpha^{'}u_{0}^{2}-2D_{0}D_{1}u_{0}$ (S27)

$\epsilon^{2}$ order equation:

${D_{0}}^{2}u_{2}+u_{2}=-2D_{0}D_{1}u_{1}-({2D}_{0}D_{2}+D_{1}^{2}{)u}_{0}-Q^{'-1}D_{0}u_{0}-\eta^{'}u_{0}^{2}D_{0}u_{0}^{2}-2\alpha^{'}u_{0}u_{1}-\beta^{'}u_{0}^{3}+\lambda^{'}cos \left( 2\omega\tau\right)u_{0}+h^{'}cos \left( \omega\tau+\varphi\right)$ (S28)

where $Q^{-1}={\epsilon^{2}Q}^{'-1}$, $\alpha=\epsilon\alpha^{'}$, $\beta=\epsilon^{2}\beta^{'}$, $\eta=\epsilon^{2}\eta^{'}$, ${\lambda=\epsilon^{2}\lambda}^{'}$, and ${h=\epsilon^{2}h}^{'}$.

From the $\epsilon^{0}$ order equation, the first order approximation of the solutions can be obtained as

$u_{0}=A\left( T_{1},T_{2} \right)e^{iT_{0}}+c.c.$ (S29)

where $c.c.$ represents the complex conjugate of the preceding term. By substituting $u_{0}$ into the $\epsilon^{1}$ order equation and eliminating the secular term, we get $D_{1}A=0$. Solving the equation describing the second-order approximation of the solution $u_{1}$ yields

$u_{1}=\frac{{\alpha^{'}A}^{2}}{3}e^{i2T_{0}}-\alpha^{'}\left| A \right|^{2}+c.c.$ (S30)

Substituting $u_{0}$ and $u_{1}$ into the $\epsilon^{2}$ order equation, eliminating the secular terms, and introducing a detuning parameter $\omega={1+\epsilon}^{2}\sigma$, we get

${iQ}^{'-1}A+i{2D}_{2}A{+D}_{1}^{2}A-\frac{10\alpha^{'2}\left| A \right|^{2}A}{3}+3\beta^{'}\left| A \right|^{2}A+i\eta^{'}\left| A \right|^{2}A-\frac{\lambda^{'}}{2}A^{*}e^{i{2\epsilon}^{2}\sigma T_{0}}-\frac{h^{'}}{2}e^{i\left( \epsilon^{2}\sigma T_{0}+\varphi_{0} \right)}=0$ (S31)

Rewriting the complex amplitude $A$ in polar form ($A=\frac{1}{2}ae^{i\theta}$) and substituting $D_{1}A=0$ into Eq. (S31), we obtain

$\left( {i\frac{1}{2}Q}^{'-1}a+i\dot{a}-\dot{\theta}a-\frac{5}{12}\alpha^{'2}a^{3}+\frac{3}{8}\beta^{'}a^{3}+\frac{i}{8}\eta^{'}a^{3}-\frac{\lambda^{'}a}{4}\cos\left( {2\epsilon}^{2}\sigma T_{0}-2\theta\right)-i\frac{\lambda^{'}a}{4}\sin\left( {2\epsilon}^{2}\sigma T_{0}-2\theta\right)-\frac{h^{'}}{2}\cos\left( \epsilon^{2}\sigma T_{0}-\theta+\varphi_{0} \right)-i\frac{h^{'}}{2}\sin\left( \epsilon^{2}\sigma T_{0}-\theta+\varphi_{0} \right) \right)e^{i\theta}=0$ (S32)

where $a$ is the steady state amplitude, and $\theta$ is the phase of the motion. Eliminate $e^{i\theta}$ and separate the real and imaginary parts of Eq. (S32) as follows

$\begin{matrix} - \dot{\varphi}a=-\sigma a-\frac{5}{12}\alpha^{'2}a^{3}+\frac{3}{8}\beta^{'}a^{3}-\frac{\lambda^{'}a}{4}\cos2\varphi-\frac{h^{'}}{2}\cos\left( \varphi+\varphi_{0} \right) \\ \dot{a}={-\frac{1}{2}Q}^{'-1}a-\frac{1}{8}\eta^{'}a^{3}+\frac{\lambda^{'}a}{4}\sin2\varphi+\frac{h^{'}}{2}\sin\left( \varphi+\varphi_{0} \right) \end{matrix}$ (S33)

where $\varphi=\sigma T_{2}-\theta$. By setting $\dot{\varphi}=0$ and $\dot{a}=0$, we obtain the steady frequency-response equations of the system under the interaction of the parametric pump voltage $V_{pump}$ and the external direct drive voltage $V_{dir}$. By setting $\dot{\varphi}=0$, $\dot{a}=0$, and $h^{'}=0$, we obtain the steady frequency-response equations of the system under the pure parametric excitation. The fitted parameters of the anti-phase mode are shown in Table S1 below.

**Supplementary Table S1.** Parameters of the device.

| Parameters | | Value |
| --- | --- | --- |
| Quality factor | 4.0$\text{×}\text{10}^{\text{4}}$ | |
| Resonant frequency $\omega_{0}/2\pi$ | 235.950 kHz | |
| Nonlinear damping $\eta$ | 0.6782 | |
| Effective cubic nonlinear stiffness $\kappa^{eff}$ ($\beta-{10\alpha^{2}}/9$) | -0.6864 | |

After we obtain the dimensionless simulation results using Eq. (S33), an important step that needs to be carried out before comparing the simulation results with the experiments is the calibration of the magnitude of the resonant vibration, i.e., the conversion of the dimensionless amplitude to the amplitude in units of voltage. We obtain the dimensional simulation results based on the following steps:

Firstly, we experimentally obtain the dimensional nonlinear amplitude-frequency response of the resonator under the pure parametric pump ($V_{pump}$=2 V and $V_{d}$=30 V), as shown in the dotted line in Fig. 1c in the main text.

Secondly, we obtain the dimensionless amplitude-response by solving Eq. (S33) in Supplementary Section 1. The parameters, such as parametric pump voltage and DC bias are consistent with those used in the experiments. The nonlinear damping and effective nonlinear stiffness are first set to zero. The effective mass is obtained according to the results in this article^5^.

Thirdly, based on the conversion factor $\mathfrak{R}_{c}=\frac{X_{exp}}{X_{the}}=\frac{\sqrt{2}R\omega_{0}\varepsilon_{0}SV_{d}}{2d_{0}}$ between the experimental results in voltage units and the dimensionless simulation results^6^ ($X_{exp}$ is the amplitude in voltage units, $X_{the}$ is the dimensionless theoretical amplitude, $R$ is the amplification of the trans-impedance amplifier, $\omega_{0}$ is the resonant frequency, $\varepsilon_{0}$ is the dielectric constant in vacuum, $S$ is the effective area of the capacitance, $V_{d}$ is the DC bias applied on the sensing electrode, and $d_{0}$ is the gap width of the capacitance), we convert the dimensionless theoretical amplitude-frequency response into the response in voltage units. The dimensionless resonant frequency is then restored to dimensional one by multiplying $\omega_{0}$.

Fourthly, since nonlinear damping and nonlinear stiffness only change the peak amplitude and curvature of the response respectively, the nonlinear damping is fitted by manually adjusting the nonlinear damping until the peak amplitude of the theoretical amplitude-frequency response in voltage units coincides with the experimental results, and the effective nonlinear stiffness is fitted by manually adjusting the effective nonlinear stiffness until the curvature/hysteresis interval of the theoretical curve coincides with the measured response.

The conversion factor $\mathfrak{R}_{c}$ is calculated as 0.37967323782266 V. All the parameters are listed in Table S1 above. Fig. 1c in the main text shows the comparison between the theoretical results and the experimental data after amplitude calibration.

**Supplementary Section 2. Theoretical analysis of the sensitivity enhancement mechanism for the symmetry breaking sensor**

In order to obtain the analytical expression of detection sensitivity, we recast Eq. (S21) and use the averaging method for deriving the differential equations of slow flow in terms of $p=acos(\psi)$ and $q=asin(\psi)$ where $a$ and $\psi$ are the slowly-varying polar coordinate representing the amplitude and phase of the transverse motion $u$. Additionally, we introduce a detuning parameter $\delta=1-\omega^{2}$ for simplicity. Eq. (S21) is then transformed to the following form^7^:

$z=\dot{u}$ (S34)

$\dot{z}+\omega^{2}u=f\left( u,z,\tau\right)$ (S35)

with

$f\left( u,z,\tau\right)=-\delta u-\frac{1}{Q}z-\kappa^{eff}u^{3}-\eta u^{2}z+\lambda\cos\left( 2\omega\tau\right)u+hcos\left( \omega\tau+\varphi_{0} \right)$ (S36)

where only the effective cubic nonlinear stiffness $\kappa^{eff}=\beta-{10\alpha^{2}}/9$ is considered to simplify analytical calculations. Van der Pol transformation is performed to set the above equations in slowly varying form:

$\binom{u}{z}=\binom{\begin{matrix} cos\omega\tau& -sin\omega\tau\end{matrix}}{\begin{matrix} -\omega sin\omega\tau& -\omega cos\omega\tau\end{matrix}}\binom{p}{q}$ (S37)

which must satisfy the following equation of constraint:

$0=\dot{p}cos\omega\tau-\dot{q}sin\omega\tau$ (S38)

We substitute Eq. (S37) into Eq. (S35), impose the constraint in Eq. (S38), and then average $\dot{p}$ and $\dot{q}$ over one whole period of vibration. The slow time equations for $p$ and $q$ can be obtained as

$\dot{p}=-\frac{1}{2\omega}\left( \frac{\omega}{Q}p+\left( \delta+\frac{\lambda}{2} \right)q+\frac{3}{4}\kappa^{eff}\left( p^{2}+q^{2} \right)q+\frac{\omega\eta}{4}\left( p^{2}+q^{2} \right)p-hsin\varphi_{0} \right)$ (S39)

$\dot{q}=-\frac{1}{2\omega}\left( \frac{\omega}{Q}q+\left( -\delta+\frac{\lambda}{2} \right)p-\frac{3}{4}\kappa^{eff}\left( p^{2}+q^{2} \right)p+\frac{\omega\eta}{4}\left( p^{2}+q^{2} \right)q+hcos\varphi_{0} \right)$ (S40)

Next, we will give a detailed description of the procedures for obtaining the phase portrait: First, we equate the horizontal axis $p$ and the vertical axis $q$ into N segments, thus obtaining N × N initial points. We use these N × N points as the initial states for numerical integration, respectively. Through numerical integration (customized ode45 program), we obtain the evolution pattern of the theoretical solution on the time scale at each initial state (similar to the orange manifold in Fig. 7d). The evolutionary trend of the system at each initial state can be obtained by drawing a tangent to the evolution pattern at each initial value. Finally, we plot the phase portrait with each initial state as the starting point and the evolutionary trend at each initial value as the direction the arrow is pointing.

The coupled slow flow is analytically unsolvable. To facilitate subsequent analytical analysis, we set $\dot{p}$ and $\dot{q}$ both equal to 0 without considering the transient process. The explicit expression of amplitude response, $a={(p^{2}+q^{2})}^{1/2}$, can then be obtained through solving Eqs. (S39) and (S40) as

$a^{2}\left[ \left( \frac{\omega}{Q}+\frac{\eta{\omega a}^{2}}{4} \right)^{2}-\left( \frac{\lambda}{2} \right)^{2}+\left( \delta+\frac{3\kappa^{eff}}{4}a^{2} \right)^{2} \right]^{2}=h^{2}\left[ \left( \frac{\omega}{Q}+\frac{\eta{\omega a}^{2}}{4} \right)^{2}+\left( \frac{\lambda}{2} \right)^{2}+\left( \delta+\frac{3\kappa^{eff}}{4}a^{2} \right)^{2}+\lambda\left( \delta+\frac{3\kappa^{eff}}{4}a^{2} \right)cos2\varphi_{0}+\lambda\left( \frac{\omega}{Q}+\frac{\eta{\omega a}^{2}}{4} \right)sin2\varphi_{0} \right]$ (S41)

From Eq. (S41), we can conclude that the nonlinear stiffness only appears together with the frequency detuning parameter as $\delta+\frac{3\kappa^{eff}}{4}a^{2}$, indicating that the nonlinear stiffness only bends the resonance curve without affecting the amplitude motion^8^. Thus, the backbone line of the system can be derived as

$\delta={-3\kappa^{eff}a^{2}}/4$ (S42)

The expressions of peak amplitude in the main branch and maximum/minimum amplitude in the isolated branch with respect to excitation intensity then can be solved through substituting Eq. (S42) into Eq. (S41). When phase difference $\varphi_{0}$ equals to $\pi/4$ where the parametric gain reaches a minimum, the implicit expression of amplitude can then be obtained in a factorized way as:

$\frac{1}{16}\left( \frac{\eta{\omega a}^{3}}{2}+\left( \lambda+\frac{2\omega}{Q} \right)a+2h \right)\left( \frac{\eta\omega a^{3}}{2}+\left( \lambda+\frac{2\omega}{Q} \right)a-2h \right)\left( \frac{\eta{\omega a}^{2}}{2}-\lambda+\frac{2\omega}{Q} \right)a^{2}=0$ (S43)

Then, $\omega=1+\frac{3\kappa^{eff}a^{2}}{8}$ is substituted into Eq. (S43) based on the fact that $\frac{1-\omega^{2}}{2}\approx\left( 1-\omega\right)$ and $\delta={-3\kappa^{eff}a^{2}}/4$ when the frequency detuning is much smaller than the resonant frequency. The expression of peak amplitude are obtained as

$\frac{1}{16}\left[ \frac{\eta a^{3}}{2}\left( 1+\frac{3\kappa^{eff}a^{2}}{8} \right)+\left( \lambda+\frac{2}{Q}\left( 1+\frac{3\kappa^{eff}a^{2}}{8} \right) \right)a+2h \right]\left[ \frac{\eta a^{3}}{2}\left( 1+\frac{3\kappa^{eff}a^{2}}{8} \right)+\left( \lambda+\frac{2}{Q}\left( 1+\frac{3\kappa^{eff}a^{2}}{8} \right) \right)a-2h \right]\left[ \frac{\eta a^{2}}{2}\left( 1+\frac{3\kappa^{eff}a^{2}}{8} \right)-\lambda+\frac{2}{Q}\left( 1+\frac{3\kappa^{eff}a^{2}}{8} \right) \right]a^{2}=0$ (S44)

The first factor has up to two positive solutions, corresponding to the maximum and minimum values of the isolated response branch as a function of the excitation intensity. The second factor shows the relationship between the peak amplitude of the main response branch and the excitation intensity, with only one positive solution at a specific excitation. The solution to the third factor has no physical meaning. Based on these, the implicit expressions of the peak and valley amplitude of the isolated branch $a_{I}$ and the peak amplitude of the main branch $a_{M}$ are derived as follows:

$-\frac{\eta a_{I}^{3}}{2}\left( 1+\frac{3\kappa^{eff}a_{I}^{2}}{8} \right)+\left( \lambda+\frac{2}{Q}\left( 1+\frac{3\kappa^{eff}a_{I}^{2}}{8} \right) \right)a_{I}=2h$ (S45)

$\frac{\eta a_{M}^{3}}{2}\left( 1+\frac{3\kappa^{eff}a_{M}^{2}}{8} \right)+\left( \lambda+\frac{2}{Q}\left( 1+\frac{3\kappa^{eff}a_{M}^{2}}{8} \right) \right)a_{M}=2h$ (S46)

The bifurcation frequencies then can be obtained by combing Eqs. (S45) and (S46) with the backbone line formula Eq. (S42). We plot the theoretical relationship between the frequencies of the bifurcation points and the input charge (in form of DC voltage $V_{e}$) in Fig. 8a in the main text to interpret the sensitivity enhancement scheme.

**Supplementary Section 3. Details of the micromechanical resonator and experimental setup**

The device under test was originally designed with a pair of force amplifiers and two gate capacitors in order to amplify the electrostatic force. During experiments, no charge was applied to the charge-input gate electrode so that the device can be regarded as a simple double-ended tuning fork. The complete structure is shown in Fig. S2. The main dimensions of the structure are listed in Table S2.


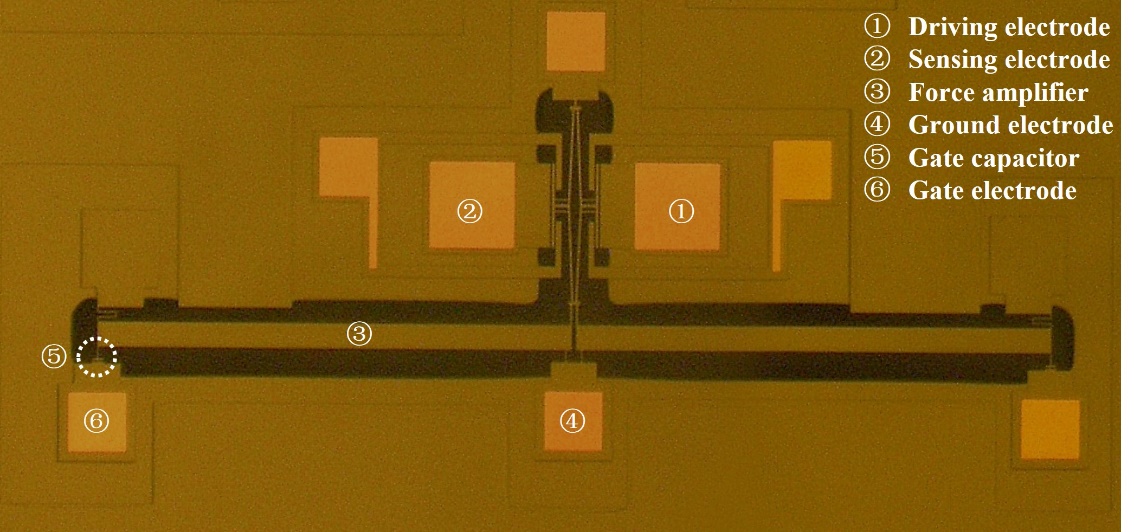


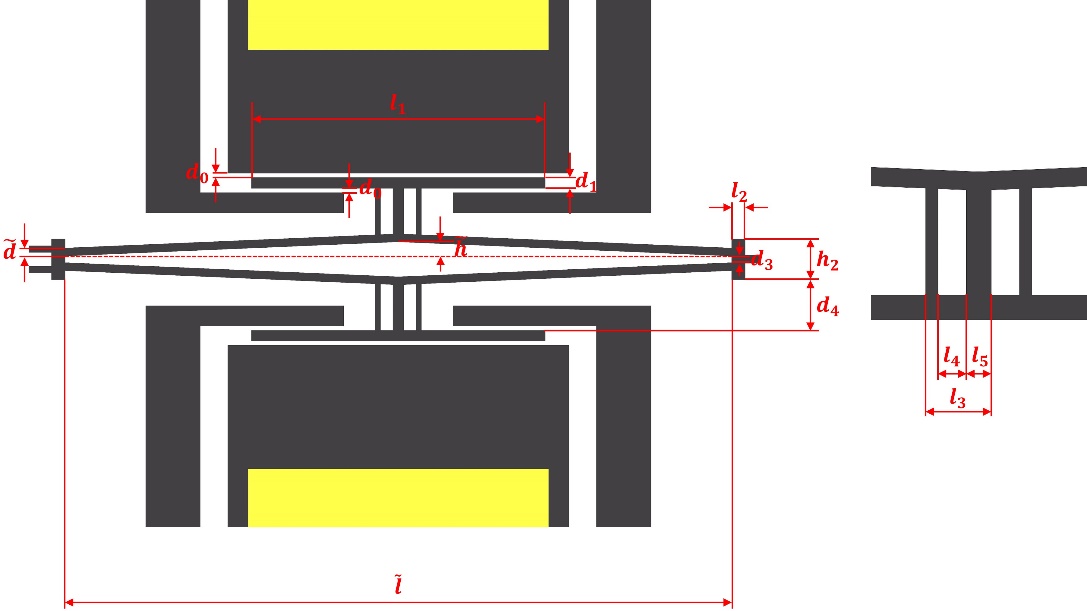


**Supplementary Figure S2.** Microscopic image of the micromechanical resonator and the main dimensions of the structure.

**Supplementary Table S2.** Main dimensions of the device.

| Parameters | | Value (μm) |
| --- | --- | --- |
| $\tilde{l}$ | 487.9 | |
| $\tilde{d}$ | 6 | |
| $d_{0}$ | 3 | |
| $l_{1}$ | 215.3 | |
| $d_{1}$ | 8 | |
| $l_{2}$ | 10 | |
| $h_{2}$ | 30 | |
| $d_{3}$ | 4.19 | |
| $d_{4}$ | 38.8 | |
| $l_{3}$ | 20.9 | |
| $l_{4}$ | 9 | |
| $l_{5}$ | 7.9 | |


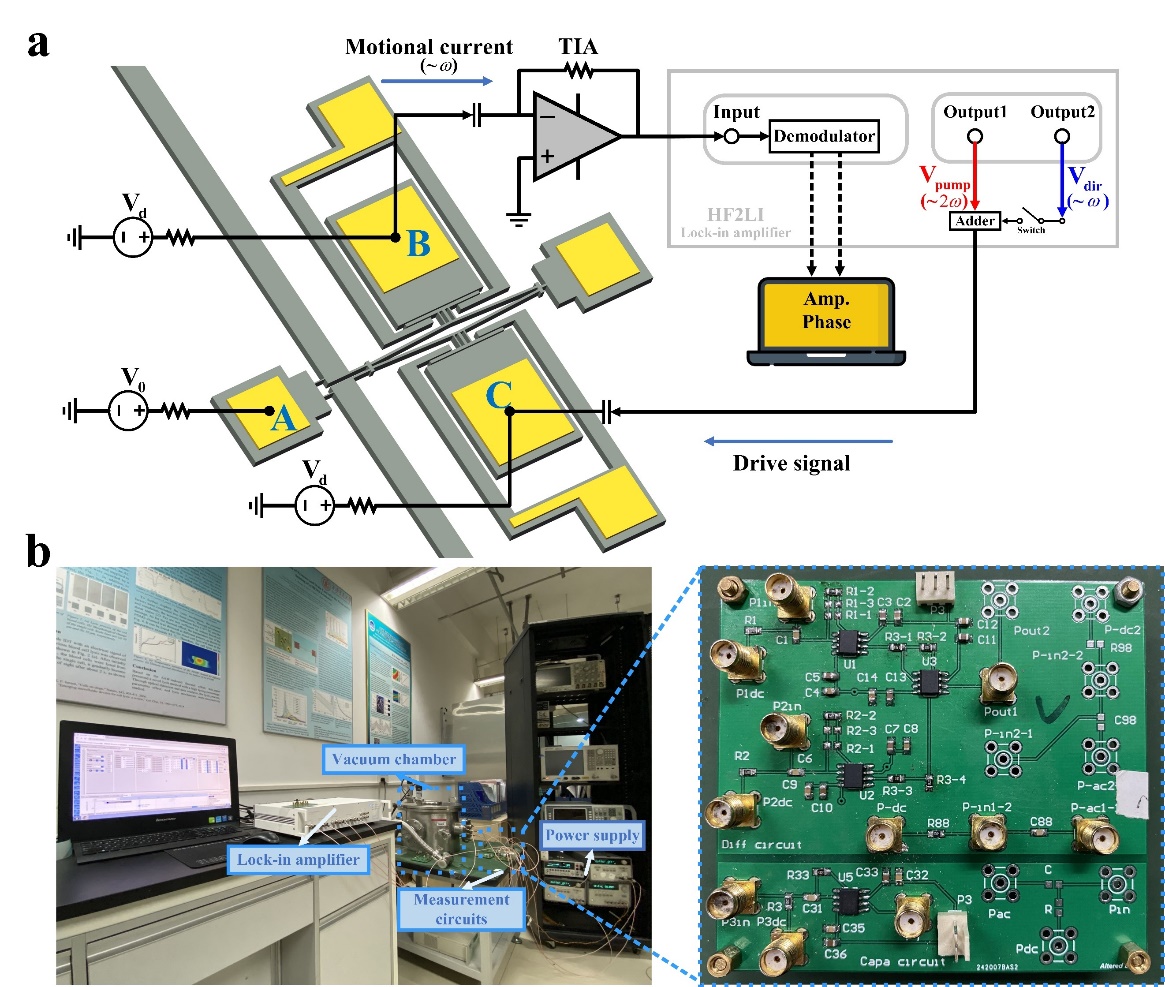


**Supplementary Figure S3.** The open-loop experimental setup used for measuring the amplitude-frequency responses of the resonator. **a** The schematic of the open-loop circuit. **b** Experimental equipment and environment.

The open-loop circuit used to measure the amplitude-frequency responses of the device is presented in Fig. S3(a). A low noise power supply was used to apply the desired DC driving voltage signal $V_{d}$ and a DC bias $V_{0}$. The DC bias $V_{0}=0$ was applied to electrode A, thereby grounding the resonator body. The Zurich Instruments lock-in amplifier was used to apply the desired AC driving voltage and extract the output signal in combination with a transimpedance amplifier (TIA). The parametric pump voltage $V_{pump}$ with a frequency near twice the resonant frequency (denoted by 2$\omega$) and the direct external drive voltage $V_{dir}$ with a frequency near the resonant frequency (denoted by $\omega$) are generated through Output1 port and Output2 port of the HF2LI lock-in amplifier, respectively. The frequencies of both AC voltage signals are controlled by the same NCO (numerically controlled oscillator) inside the lock-in amplifier. The NCO output with frequency $\omega$ is amplified to provide the desired $V_{dir}$ at the Output2 port. As for the parametric pump voltage $V_{pump}$, the NCO output with frequency $\omega$ is amplified and frequency doubled to provide the desired $V_{pump}$ at the Output1 port.

The phase difference between the $V_{pump}$ and $V_{dir}$ can be manually adjusted in the host computer. When the switch is closed, the two AC driving voltage can be added together by the built-in adder of the lock-in amplifier, allowing for separate external direct excitation ($V_{dir}\neq$0 and $V_{pump}=0$), separate parametric excitation ($V_{dir}=$0 and $V_{pump}\neq0$) or combined excitation ($V_{dir}\neq$0 and $V_{pump}\neq0$). With the switch being closed, the resonator was electrostatically excited by applying the combined voltage of $V(t)=V_{ac}+V_{d}$ to electrode C, where $V_{ac}=V_{pump}+V_{dir}$ and $V_{d}$ is the DC driving voltage.

The motion of the resonator was detected via capacitive sensing method. The capacitance value of the sensing capacitor $C_{s}$ consisting of the resonator body and the fixed electrode B changes during the vibration of the resonator body, resulting in a change in charge. A change in charge causes a motional current. The expression of the motional current is

$$\begin{matrix} & i=\frac{\partial Q_{s}}{\partial t}=V\frac{\partial C_{s}}{\partial t} \\ & =V\frac{\partial C_{s}}{\partial x}\frac{\partial x}{\partial t} \\ & =V\left( \frac{\varepsilon ab}{d_{0}^{2}}-\frac{2\varepsilon ab}{d_{0}^{3}}x+\frac{3\varepsilon ab}{d_{0}^{4}}x^{2}-\frac{4\varepsilon ab}{d_{0}^{5}}x^{3}-... \right)\dot{x} \\ & \approx V\frac{\varepsilon ab}{d_{0}^{2}}\dot{x} \end{matrix}$$

where $V$ is the potential difference between the resonator body and the fixed electrode B and its value equals $V_{d}$ since the bias voltage on the resonator is 0 V, $x$ is the displacement, $\dot{x}$ is the velocity, $t$ is the time, $Q_{s}$ is the charge, $\varepsilon$ is the dielectric constant, $ab$ is the effective area of the capacitor, and $d_{0}$ is the gap width. By ignoring the nonlinear term of $x$, the motion current is proportional to the velocity.

The motional current representing the magnitude of the mechanical motion is amplified by the transimpedance amplifier and converted into a voltage signal. The amplified signal is then demodulated by the built-in demodulator in the lock-in amplifier to extract the amplitude and phase with respect to the frequency of the NCO at $\omega$. The demodulated amplitude-frequency response and phase-frequency response are displayed and stored by the host computer.


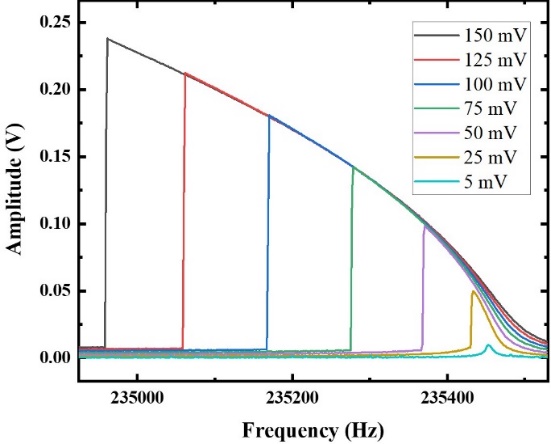


**Supplementary Figure S4.** The open-loop amplitude responses of the device under only direct drive.

The responses of the device with only direct drive voltage $V_{dir}$ is shown in Fig. S4. The DC bias $V_{d}$ is set to 40 V, and the direct drive voltage $V_{dir}$ is increased from 5 mV to 150 mV. With the increasing of $V_{dir}$, the amplitude response gradually transforms from linear to nonlinear, exhibiting a negative Duffing-like nonlinearity. It is worth noting that the amplitude-frequency responses shown in Fig. S4 were obtained using another device of the same dimensions as in the main text.


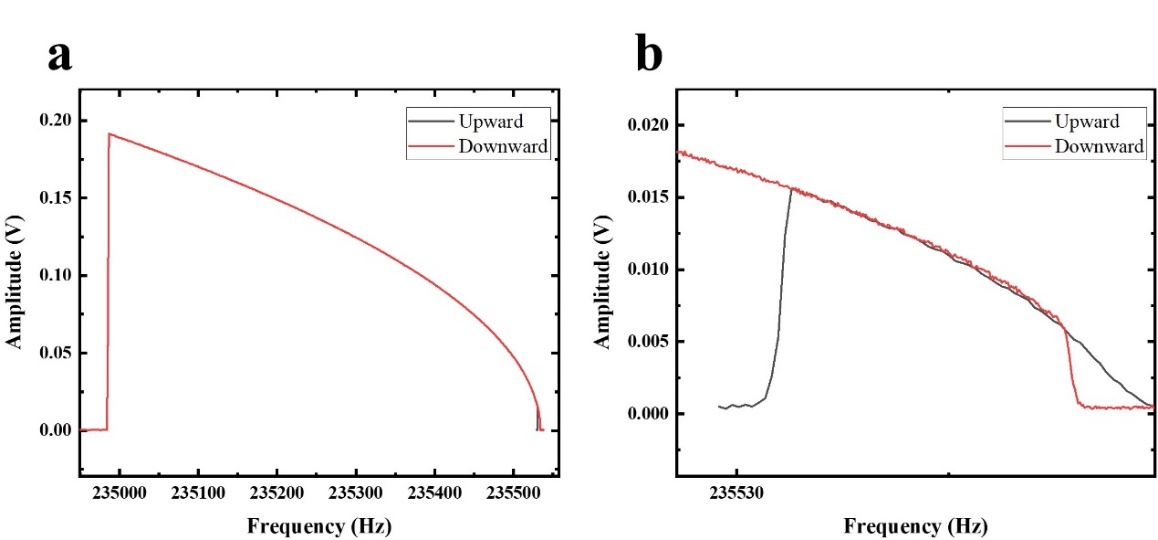


**Supplementary Figure S5.** **a** Amplitude-frequency responses of the device obtained by downward and upward frequency sweep. **b** Zoom-in of (**a**).

As shown in Fig. S5(a), the red solid line and black solid line are the parametric responses obtained by downward and upward frequency sweep respectively when the DC driving voltage and parametric pump voltage are equal to 30 V and 1 V, respectively. Fig. S5(b) is the zoom-in of the amplitude responses. The response obtained by upward frequency sweep is very small compared to the response obtained by downward sweep.

**Supplementary Section 4. Closed-loop experimental configuration and limitations of the phase-locked loop**

The closed-loop circuit used to conduct the real-time charge detection is presented in Fig. S6(a). The phase-locked loop consists of a phase detector, a bandpass filter, a PID controller and a numerically controlled oscillator. We investigate the impact of PLL parameters on charge detection performance. By setting the bandwidth of PLL to 30 Hz, we achieve a high charge sensitivity of up to 39.5 $\text{ppm∙}\text{fC}^{\text{-1}}$ (6.33 ppb/e), as shown by the orange dots in Fig. S6(c). The minimum Allan deviation is 0.19138 ppm in this case, corresponding to a charge resolution of 30.2 electrons. However, the increase in measurement sensitivity comes at the expense of frequency stability.


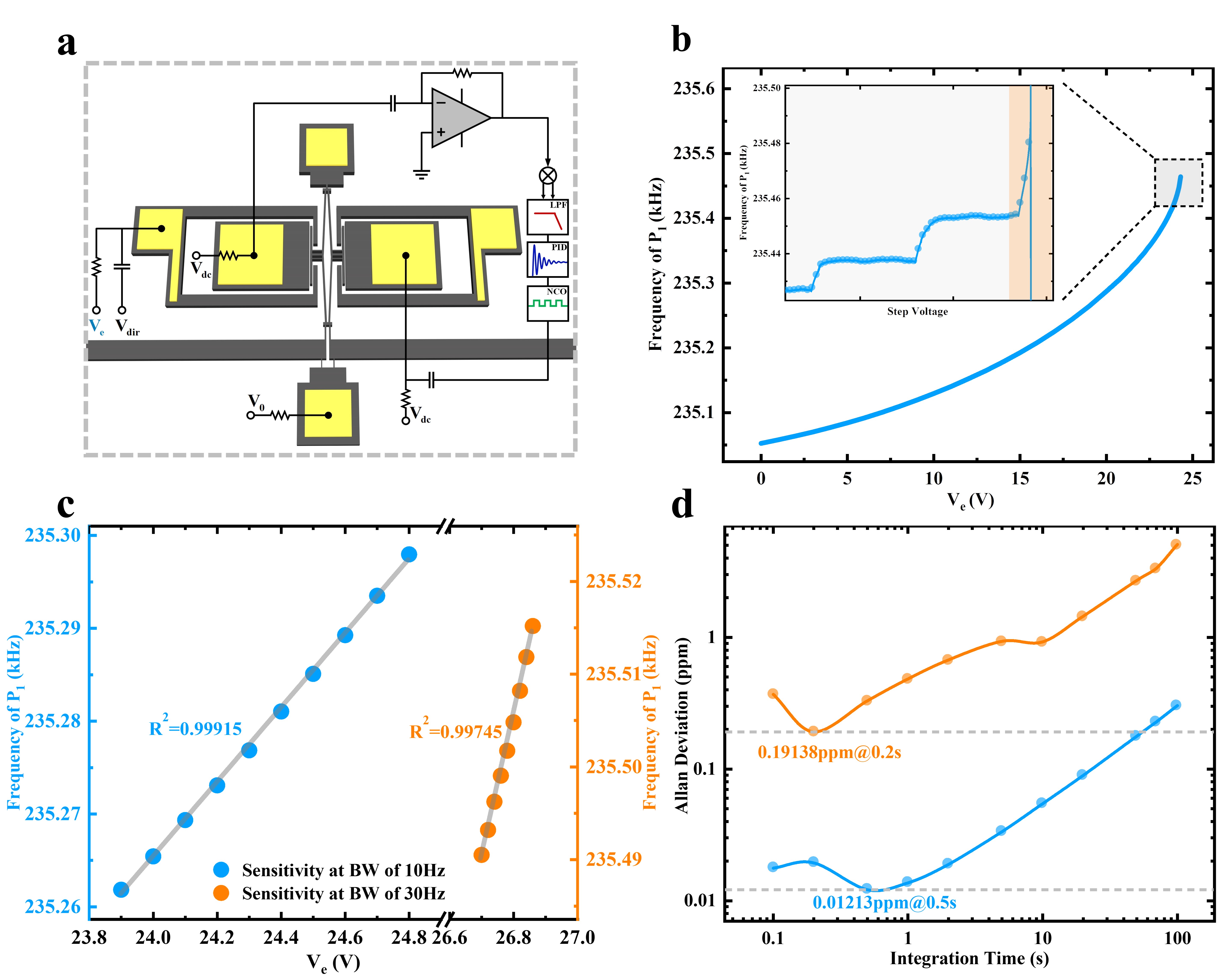


**Supplementary Figure S6.** Closed-loop configuration and the impact of phase-locked loop parameters on sensing performance.

The blue dots in Fig. S6(c) show the charge sensitivity of our sensing scheme at a PLL bandwidth of 10 Hz; the blue dot line in Fig. S6(d) shows the Allan deviation of the oscillation frequency at this PLL bandwidth. Although the reduction in PLL bandwidth degrades the frequency tracking capacity of the PLL, resulting in a lower measurement sensitivity of 10.3 $\text{ppm∙}{\text{f}\text{C}}^{\text{-1}}$, the minimum Allan deviation reduces by a factor of 15.78 (from 0.19138 ppm to 0.01213ppm). Therefore, the measurement resolution is improved from 30.2 electrons to 7.34 electrons compared to the case with a PLL bandwidth of 30 Hz.

The mutual constraints between the frequency tracking capability and the frequency stability are owing to the fact that PLL will filter out the fluctuations whose time scale are faster than the corner frequency of the transfer function to optimize the frequency stability^9^. A smaller PLL bandwidth implies better frequency stability but a longer time to track the frequency and reach a steady state. The degraded frequency tracking ability causes the phase locking to be more prone to fail when approaching the point where two saddle-node bifurcation points annihilate, as shown in the inset of Fig. S6(b). Therefore, Better frequency tracking performance at a larger bandwidth (30 Hz) can maintain stable self-sustained oscillation even at an extremely large frequency variation, resulting in a higher measurement sensitivity, while greater frequency stability can be achieved at a relatively small PLL bandwidth (10 Hz). Therefore, we can achieve a higher measurement resolution of 7.34 electrons or a higher measurement sensitivity of up to 6.33 ppb/e (39.5 $\text{ppm∙}{\text{f}\text{C}}^{\text{-1}}$), depending on the PLL bandwidth.

**Supplementary Section 5. Comparison with the state-of-the-art resonator-based charge sensors**

We compare the charge sensor from this work, in terms of resolution and sensitivity to the resonator-based charge sensors reported in literature in recent years, shown in Table S3. The resonator-based charge sensors can be divided into two categories, namely resonant charge sensors and mode-localized charge sensors. Resonant charge sensors achieve sensing by tracking frequency shift, while mode-localized sensors achieve sensing by tracking changes in the amplitude ratio of the coupled resonators.

The sensor in this work has a charge sensitivity of 39.5 ppm/*f*C, exceeding the best reported result of 22.532 ppm/*f*C when compared to other counterparts of resonant charge sensors. Although the mode-localized charge sensors have higher sensitivity, the charge resolution of the mode-localized sensors is generally worse than that of resonant sensors owing to their poor amplitude stability.

**Supplementary Table S3.** Comparisons of the state-of-the-art resonator-based charge sensors.

| Year [Ref.] | | Size($\text{μm}^{\text{3}}$) | Mechanism | Resolution(e) | Sensitivity |
| --- | --- | --- | --- | --- | --- |
| 2017^10^ | 1500×1000×25 | | Resonant | 203750 | 8.008 ppm/*f*C |
| 2018^11^ | 1425×1050×25 | | Resonant | 16250 | 9.325 ppm/*f*C |
| 2020^12^ | 127.3×127.3×25 | | Resonant | 0.17 | 22.532 ppm/*f*C |
| 2016^13^ | \ | | Mode localization | 7900 | 11,213.4 ppm/*f*C |
| 2018^14^ | 1280×2700×30 | | Mode localization | 9.21 | 2,280,858 ppm/*f*C |
| 2024^15^ | 350×103×25 | | Mode localization | 14 | 76,313.28 ppm/*f*C |
| 2020^16^ | 350×103×25 | | Resonant (Internal resonance) | 0.197 | 13.154 ppm/*f*C |
| 2023^17^ | 550×320×25 | | Resonant (Internal resonance) | 63 | 19.295 ppm/*f*C |
| **This work** | **487.9×215.3×25** | | **Resonant (Symmetry breaking)** | **7.34** | **39.5 ppm/*f*C** |

**Note**: The Size in this table represents the dimension of the vibrational part of the device.

**Supplementary references**

1 Chen, Y. On the vibration of beams or rods carrying a concentrated mass. (1963).

2 Younis, M. I. *MEMS linear and nonlinear statics and dynamics*. Vol. 20 (Springer Science & Business Media, 2011).

3 Chen, Y. On the Vibration of Beams or Rods Carrying a Concentrated Mass. *Journal of Applied Mechanics* **30**, 310-311, doi:10.1115/1.3636537 (1963).

4 Younis, M. I. MEMS Linear and Nonlinear Statics and Dynamics. (2011).

5 Hauer, B., Doolin, C., Beach, K. & Davis, J. A general procedure for thermomechanical calibration of nano/micro-mechanical resonators. *Annals of Physics* **339**, 181-207 (2013).

6 Agarwal, M. *et al.* Scaling of amplitude-frequency-dependence nonlinearities in electrostatically transduced microresonators. *Journal of Applied Physics* **102** (2007).

7 Batista, A. A. Cooling, heating, and thermal noise squeezing in a parametrically driven resonator. *Journal of Statistical Mechanics: Theory and Experiment* **2011**, doi:10.1088/1742-5468/2011/02/p02007 (2011).

8 Li, D. & Shaw, S. W. The effects of nonlinear damping on degenerate parametric amplification. *Nonlinear Dynamics* **102**, 2433-2452, doi:10.1007/s11071-020-06090-8 (2020).

9 Roy, S. K., Sauer, V. T. K., Westwood-Bachman, J. N., Venkatasubramanian, A. & Hiebert, W. K. Improving mechanical sensor performance through larger damping. *Science* **360**, doi:10.1126/science.aar5220 (2018).

10 Chen, D., Zhao, J., Wang, Y. & Xie, J. An electrostatic charge sensor based on micro resonator with sensing scheme of effective stiffness perturbation. *Journal of Micromechanics and Microengineering* **27**, doi:10.1088/1361-6439/aa6b41 (2017).

11 Chen, D., Zhao, J., Wang, Y., Xu, Z. & Xie, J. Sensitivity manipulation on micro-machined resonant electrometer toward high resolution and large dynamic range. *Applied Physics Letters* **112**, doi:10.1063/1.5009276 (2018).

12 Chen, D. *et al.* Ultrasensitive Resonant Electrometry Utilizing Micromechanical Oscillators. *Physical Review Applied* **14**, doi:10.1103/PhysRevApplied.14.014001 (2020).

13 Zhang, H., Huang, J., Yuan, W. & Chang, H. A High-Sensitivity Micromechanical Electrometer Based on Mode Localization of Two Degree-of-Freedom Weakly Coupled Resonators. *Journal of Microelectromechanical Systems* **25**, 937-946, doi:10.1109/jmems.2016.2598780 (2016).

14 Yang, J., Kang, H. & Chang, H. in *2018 IEEE Micro Electro Mechanical Systems (MEMS).* 67-70 (IEEE).

15 Liu, Z. *et al.* Nonlinearity enhanced mode localization in two coupled MEMS resonators. *International Journal of Mechanical Sciences* **271**, doi:10.1016/j.ijmecsci.2024.109133 (2024).

16 Wang, X., Wei, X., Pu, D. & Huan, R. Single-electron detection utilizing coupled nonlinear microresonators. *Microsyst Nanoeng* **6**, 78, doi:10.1038/s41378-020-00192-4 (2020).

17 Qiao, Y. *et al.* Frequency unlocking-based MEMS bifurcation sensors. *Microsyst Nanoeng* **9**, 58, doi:10.1038/s41378-023-00522-2 (2023).
